# Thorium- and uranium-azide reductions: a transient dithorium-nitride *versus* isolable diuranium-nitrides†
†Electronic supplementary information (ESI) available: Full experimental and computational details. CCDC 1868610–1868615. For ESI and crystallographic data in CIF or other electronic format see DOI: 10.1039/c8sc05473h


**DOI:** 10.1039/c8sc05473h

**Published:** 2019-02-23

**Authors:** Jingzhen Du, David M. King, Lucile Chatelain, Erli Lu, Floriana Tuna, Eric J. L. McInnes, Ashley J. Wooles, Laurent Maron, Stephen T. Liddle

**Affiliations:** a School of Chemistry , The University of Manchester , Oxford Road , Manchester , M13 9PL , UK . Email: steve.liddle@manchester.ac.uk; b School of Chemistry , The University of Nottingham , University Park , Nottingham , NG7 2RD , UK; c LPCNO , CNRS , INSA , Université Paul Sabatier , 135 Avenue de Rangueil , Toulouse 31077 , France . Email: laurent.maron@irsamc.ups-tlse.fr

## Abstract

Molecular uranium-nitrides are now well known, but there are no isolable molecular thorium-nitrides outside of cryogenic matrix isolation experiments. We report that treatment of [M(Tren^DMBS^)(I)] (M = U, **1**; Th, **2**; Tren^DMBS^ = {N(CH_2_CH_2_NSiMe_2_Bu^*t*^)_3_}^3–^) with NaN_3_ or KN_3_, respectively, affords very rare examples of actinide molecular square and triangle complexes [{M(Tren^DMBS^)(μ-N_3_)}_*n*_] (M = U, *n* = 4, **3**; Th, *n* = 3, **4**). Chemical reduction of **3** produces [{U(Tren^DMBS^)}_2_(μ-N)][K(THF)_6_] (**5**) and [{U(Tren^DMBS^)}_2_(μ-N)] (**6**), whereas photolysis produces exclusively **6**. Complexes **5** and **6** can be reversibly inter-converted by oxidation and reduction, respectively, showing that these UNU cores are robust with no evidence for any C–H bond activations being observed. In contrast, reductions of **4** in arene or ethereal solvents gives [{Th(Tren^DMBS^)}_2_(μ-NH)] (**7**) or [{Th(Tren^DMBS^)}{Th(N[CH_2_CH_2_NSiMe_2_Bu^*t*^]_2_CH_2_CH_2_NSi[μ-CH_2_]MeBu^*t*^)}(μ-NH)][K(DME)_4_] (**8**), respectively, providing evidence unprecedented outside of matrix isolation for a transient dithorium-nitride. This suggests that thorium-nitrides are intrinsically much more reactive than uranium-nitrides, since they consistently activate C–H bonds to form rare examples of Th–N(H)–Th linkages with alkyl by-products. The conversion here of a bridging thorium(iv)-nitride to imido-alkyl combination by 1,2-addition parallels the reactivity of transient terminal uranium(iv)-nitrides, but contrasts to terminal uranium(vi)-nitrides that produce alkyl-amides by 1,1-insertion, suggesting a systematic general pattern of C–H activation chemistry for metal(iv)- *vs.* metal(vi)-nitrides. Surprisingly, computational studies reveal a σ > π energy ordering for all these bridging nitride bonds, a phenomenon for actinides only observed before in terminal uranium nitrides and uranyl with very short U–N or U–O distances.

## Abstract 2

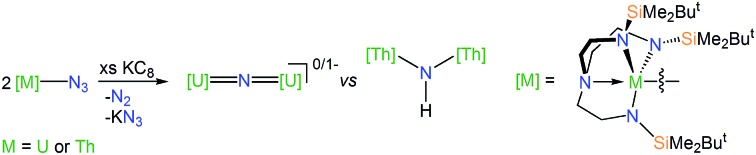
Evidence for a transient, highly reactive ThNTh nitride is presented, in contrast to uranium analogues that are stable and isolable. Surprisingly, computational studies reveal a σ > π energy ordering for all these bridging nitride bonds, a phenomenon for actinides only observed before in terminal uranium nitrides and uranyl.

## Introduction

In recent years there have been major developments in uranium-ligand multiple bonding,^1^ and arguably none more so than U–N multiple bonds.^2^ For example, significant advances include bis-, tris-, and tetrakis-imido complexes,^3^–^5^ two parent imido complexes,^6^ reductive cleavage and functionalisation of N_2_,^7^ terminal N_2_ and NO complexes,^8^ and the emergence of isolable uranium-nitrides.^9^–^11^ Such studies are motivated by a desire to prepare actinide congeners of linkages known for decades in the d-block in order to better understand covalency in actinide chemical bonding and to map out intrinsic reactivity trends;^12^ however, equivalent metal–ligand bonds need to be compared with different actinide elements to elucidate periodic trends. In that regard, thorium analogues demand attention, but there are few mono-imido and bis-imido derivatives, and no parent imidos.^13^ There are no isolable molecular thorium-nitrides to date, but under cryogenic matrix isolation conditions elegant species such as ThN, F_3_ThN, NThN, Th(N)_2_Th, and NThO have been reported.^14^ A transient zero-valent thorium synthon produced a Th–NH_2_ linkage from N_2_,^15^ but it is not known whether this transformation involves a transient thorium-nitride or if a conventional biomimetic-type (H^+^/e^–^) pathway is followed.

Building on our prior work on terminal uranium-nitrides,^11^ we have reported complexes containing parent terminal U = EH (E = N, P, As) and bridging UP(H)U, UPU, and UAsK_2_ linkages.6a,16a,b,e For thorium, we have prepared complexes exhibiting parent terminal Th=PH, and bridging ThP(H)Th, ThAs(H)Th, ThAs(H)K, ThPTh and ThAsTh units.16c,d We previously observed that bridging ThPTh and ThAsTh linkages are stable and isolable in triamidoamine derivatives, but conversely UPU and UAsK_2_ linkages are highly unstable and readily decompose. In contrast, most bridging UNU units seem to be relatively stable,10b,f,g,j,k though none are known with triamidoamine ancillary ligands. Thus, how ThNTh units would fit into these stability patterns is unknown and cannot be predicted with any certainty. So, we have initially sought to prepare dithorium-nitrides, as well as diuranium analogues for comparison, supported by triamidoamines.

Here, we report the synthesis and characterisation of two triamidoamine uranium- and thorium-azides. Despite marginal differences in the covalent radii of these metals, the uranium complex is a rare example of an actinide molecular square whereas the thorium analogue is a molecular triangle. Reduction of the uranium-azide complex generates diuranium-nitrides, with two charge states of a UNU core being accessible, and interchangeable, with no evidence of C–H activation chemistry even under photolytic conditions. However, a ThNTh complex could not be isolated. Instead, the isolation of two ThN(H)Th complexes, which is an unprecedented linkage in thorium chemistry and rare in f-block chemistry generally,7c,d suggests that a dithorium-nitride complex is transiently formed but activates any available C–H bonds, be they part of arene solvent or in the absence of arenes the triamidoamine ligand. The ThNTh unit therefore appears to be highly reactive in a triamidoamine ligand environment, and its instability contrasts to ThPTh and ThAsTh congeners and UNU analogues and also suggests a systematic pattern of metal oxidation state-dependent C–H activation reactivity for actinide-nitrides. Surprisingly, computational studies reveal a σ > π energy ordering for the bridging nitride linkages in this study, a phenomenon so far only found in terminal uranium-nitrides and uranyl complexes with very short U–N/–O distances.

## Results and discussion

### Preparation of uranium- and thorium-azide complexes

**Scheme 1:**
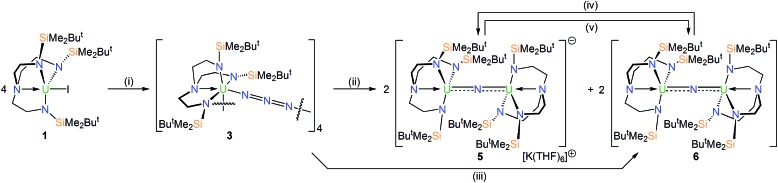
Synthesis of **3**, **5** and **6** from **1**. Reagents and conditions: (i) 4NaN_3_, THF, –4NaI; (ii) 4KC_8_, THF, –4C_8_, –2KN_3_, –2N_2_; (iii) *hν*, 125 W Hg-lamp, 7 h, toluene, –5N_2_; (iv) KC_8_, THF, –C_8_; (v) AgBPh_4_, toluene, –KBPh_4_, –Ag^0^, –6THF.

**Scheme 2:**
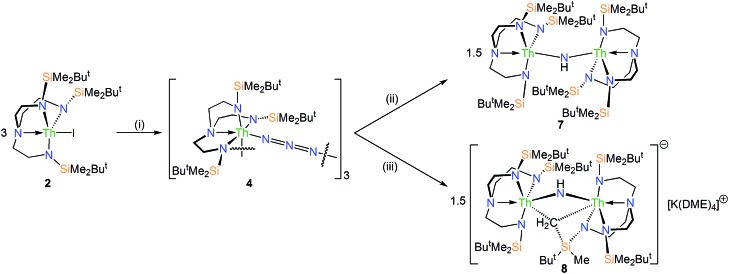
Synthesis of **4**, **7** and **8** from **2**. Reagents and conditions: (i) 3KN_3_, THF, –3KI; (ii) 3KC_8_, benzene or toluene, –3C_8_, –1.5 KN_3_, –1.5N_2_, –KCH_2_Ph or –KC_6_H_5_; (iii) 3KC_8_, THF or DME, –1.5KN_3_, –1.5N_2_.

**Fig. 1:**
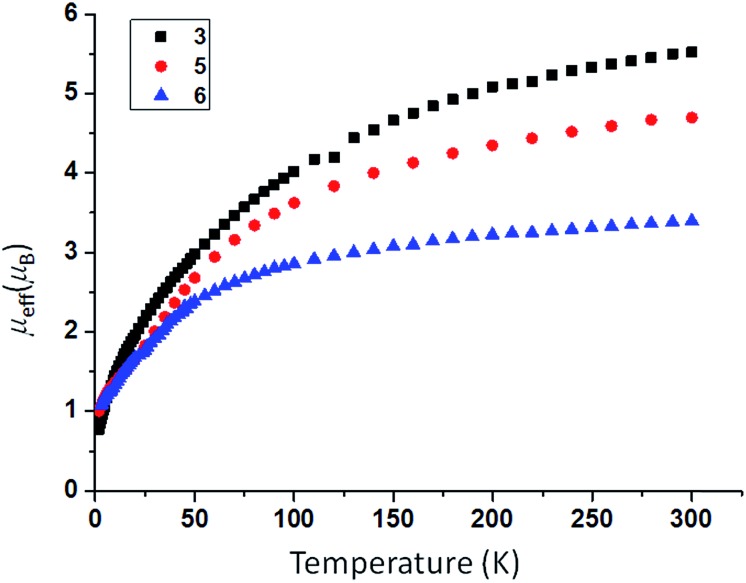
Variable temperature SQUID magnetic moment data for **3** (black squares), **5** (red circles), and **6** (blue triangles) over the range 2–300 K.

In order to prepare MNM linkages we pursued a M–N_3_ reduction approach using the Tren^DMBS^ {N(CH_2_CH_2_NSiMe_2_Bu^*t*^)_3_}^3–^ ligand as this was anticipated to be sterically open enough to allow any nitrides to bridge, whereas the bulkier Tren^TIPS^ {N(CH_2_CH_2_NSiPr^i^_3_)_3_}^3–^ variant stabilises ThPTh and ThAsTh linkages but terminal UN for uranium. Accordingly, treatment of [M(Tren^DMBS^)(I)] (M = U, **1**; Th, **2**)^17^ with excess NaN_3_ or KN_3_ affords [{M(Tren^DMBS^)(μ-N_3_)}_*n*_] (M = U, *n* = 4, **3**; Th, *n* = 3, **4**) as green-yellow and colourless crystalline solids after work-up in isolated yields of 35 and 86%, respectively, Schemes 1 and 2.^18^ The combined characterisation data support the formulations of **3** and **4**, in particular the ATR-IR spectra of **3** and **4** both exhibit strong absorptions at 2131 cm^–1^, which is characteristic of actinide-bound bridging-azide ligands.10b,c,^19^ The magnetic moment of **3**, Fig. 1,^18^ in the solid-state at 298 K is 5.52 *μ*_B_ per molecule decreasing smoothly to 0.78 *μ*_B_ at 2 K (3.29 and 0.41 *μ*_B_ per uranium ion in **3**, respectively) and tending to zero as expected for a tetrametallic U^IV^ complex, since U^IV^ usually has a magnetic singlet ground state at low temperature.^2^,^20^

**Fig. 2:**
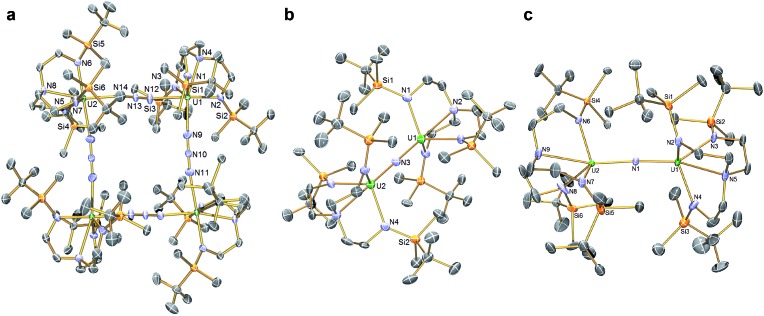
Molecular structures of (a) **3**, (b) the anion component of **5**, (c) **6**. Structures are depicted with selective symmetry-unique labels, 40% probability displacement ellipsoids, and hydrogen atoms, minor disorder components, and lattice solvent molecules are omitted for clarity.

**Fig. 3:**
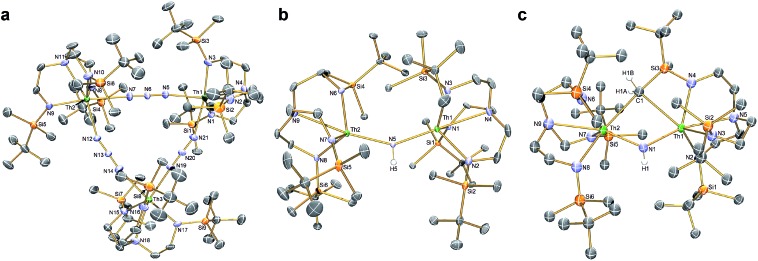
Molecular structures of (a) **4**, (b) **7**, (c) the anion component of **8**. Structures are depicted with selective labels, 40% probability displacement ellipsoids and hydrogen atoms (except N–H and H_2_CSi), minor disorder components, and lattice solvent molecules are omitted for clarity.

In order to confirm the formulations of **3** and **4** we determined their solid-state structures, Fig. 2a and 3a. Surprisingly, although **1** and **2**, and chloride analogues, are monomers, **3** and **4** are tetrameric and trimeric in the solid-state. Such molecular squares and triangles^21^ are relatively rare motifs in actinide chemistry,10b,c,^22^ since polymeric formulations tend to dominate19*f* as there is not usually a strong orbitally-driven geometric preference for M–L angles that generate squares or triangles. However, it would appear that when the *C*_3v_ symmetry of Tren^DMBS^ is lowered to *C*_s_ the cleft that opens up allows two azides to enter the coordination sphere of uranium in **3** at an approximate right angle (∼85°) whereas for the larger thorium in **4** the azides approach at a slightly more acute N–Th–N angle (∼79°), which seems to be enough to switch from tetramer to trimer. It would seem that the N–Th–N angle can close at the larger metal without as much inter-azide clashing due to longer Th–N bonds placing the azides further apart from one another, which accounts for the aggregation states of **3** and **4**. The U– and Th–N_azide_ distances in **3** and **4** are longer than in terminal azide complexes,^19^ and we note that they are longer when *trans* to a Tren^DMBS^ amide centre (**3**, 2.540(5); **4**, 2.609(8) Å av.) than amine centre (**3**, 2.425(5); **4**, 2.478(7) Å av.), possibly implying a *trans*-influence. All other bond lengths are within normal ranges and do not suggest any strong activation of the azides.

### Uranium-azide reductions and characterisation of nitride products

With **3** and **4** in-hand we investigated their reduction chemistries. The reaction of **3** with KC_8_ always produces the diuranium(iv/iv)-nitride [{U(Tren^DMBS^)}_2_(μ-N)][K(THF)_6_] (**5**) and the mixed-valence diuranium(iv/v)-nitride [{U(Tren^DMBS^)}_2_(μ-N)] (**6**) in ∼42% overall yield (total yield by uranium content, 28% **5** and 14% **6** estimated from integration of ^1^H NMR data), with concomitant elimination of N_2_ and KN_3_.^18^ The ratio of **5** : **6** varies from 77 : 23 to 50 : 50 as the KC_8_ ratio is varied from 5 to 3 equivalents but is independent of the solvent used (benzene, toluene, THF, DME). Other KC_8_ ratios gave intractable product mixtures. Complexes **5** and **6** can be separated by fractional crystallisation, however we find that **6** can be cleanly prepared in 45% isolated crystalline yield by photolysis of **3** with a 125 W Hg-lamp for 7 hours. Gratifyingly, **6**, or a known mixture of **5** and **6**, can be reduced with KC_8_ to give solely **5**, and **5** or a known mixture of **5** and **6** can be oxidised with AgBPh_4_ to give exclusively **6**, Scheme 1. Interestingly, **5** and **6** are always formed in the respective reduction and oxidation reactions, irrespective of the amounts of KC_8_ (2–8 equiv.) and AgBPh_4_ (2–3 equiv.) used, and we found no evidence for further reductions or oxidations, respectively.

**Fig. 4:**
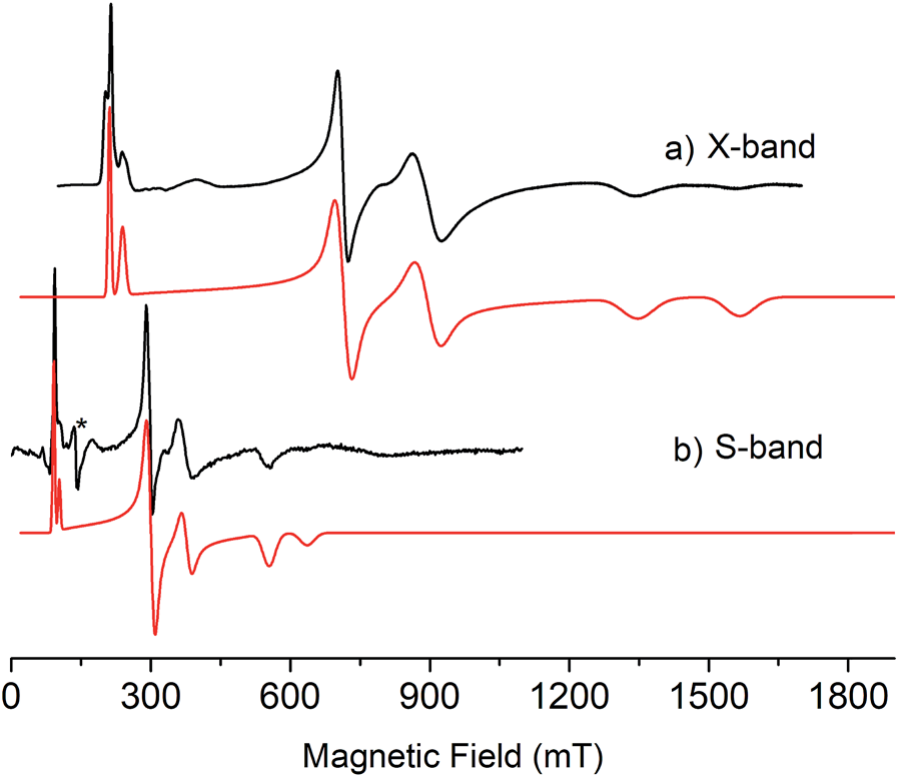
(a) X-band (9.39 GHz) and (b) S-band (3.87 GHz) EPR spectra of polycrystalline samples of **6** at 5 K. Black lines are experimental data and red lines are simulations. * = cavity signal.

**Fig. 5:**
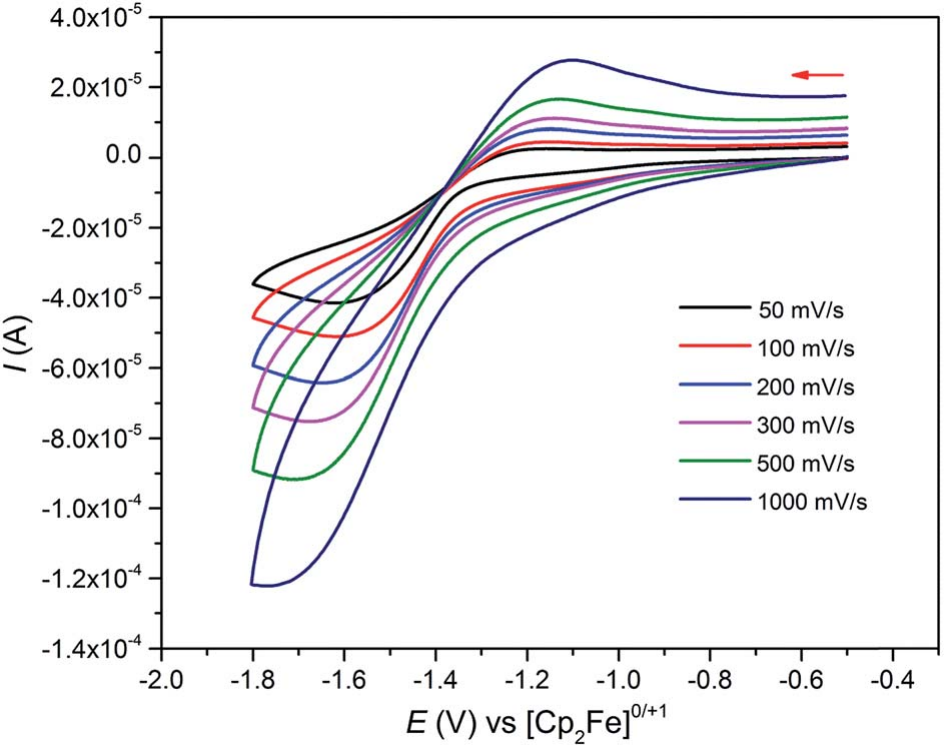
Cyclic voltammogram of 0.1 M **6** in THF at selected sweep rates (0.5 M [N(^*n*^Bu)_4_][BAr^F^_4_] supporting electrolyte) *vs.* [Fe(Cp)_2_]^+/0^ showing a single irreversible redox process. Test solutions of **5** or **6** in THF with [N(^*n*^Bu)_4_][BAr^F^_4_] under identical conditions show no evidence of stability issues.

As expected, the characterisation data for **5** and **6** are distinct, reflecting their U^IV^/U^IV^ and U^IV^/U^V^ formulations, respectively. The ^1^H NMR spectra of **5** and **6** span the ranges +96 to –34 and +25 to –13 ppm, respectively, reflecting their 5f^2/2^*vs.* 5f^2/1^ natures. The solution Evans method (298 K) gives magnetic moments of 4.0 and 3.5 *μ*_B_ per molecule of **5** and **6**, whereas the solid-state magnetic moments, Fig. 1,^18^ are 4.70 and 3.39 *μ*_B_, respectively. These values decrease to 1.00 and 1.07 *μ*_B_ at 2 K, respectively. For **5** the respective values per uranium ion are 3.39 (298 k) and 0.74 *μ*_B_ (2 K), which per ion are slightly higher than the corresponding values for **3** reflecting their nitride and azide formulations.16b,^23^ The data for **6** are consistent with its U^IV^/U^V^ combination,^2^,^20^ where the U^V^ ion has a magnetic doublet ground state at all temperatures, and anti-ferromagnetic U–U coupling is suggested by a maximum at ∼60 K in the *χ vs. T* plot of **5**.^2^,^24^,^25^ The presence of U^V^ in **6** is unequivocally confirmed by EPR spectroscopy (S- and X-bands) at 5 K, Fig. 4,^18^ which reveals two similar sets of rhombic *g*-values with *g*_eff_ = 3.13, 0.95, 0.50, and 2.70, 0.74, 0.43; these data reflect the presence of two conformational isomers in the solid-state structure of **6** due to positional disorder of three of the six SiMe_2_Bu^*t*^ groups, and we note that the effective *g*-values of spin–orbit doubles are extremely sensitive to small changes in structure.^26^ An electrochemical irreversible one-electron process at *E*_1/2_ –1.4 V (*vs.* [Fc(Cp)_2_]^0/+1^) for the [U^IV^/U^V^]/[U^IV^/U^IV^]^–^ redox couple is found, Fig. 5, which contrasts to **3** and **4** which do not exhibit any electrochemical events in the solvent-accessible window of 2.5 to –3.0 V. The chemical inter-conversion of **5** and **6** suggests the presence of robust UNU cores, as was found for [{U(NBu^*t*^[3,5-Me_2_C_6_H_3_])_3_}_2_(μ-N)]^*n*^ (*n* = +1, 0, –1) which can exist in three charge states,10*f* but the irreversible electrochemical behaviour may reflect structural changes in the U=N=U angles of **5** compared to **6** (see below).

The solid-state structures of **5** and **6** were determined, Fig. 2b and c, revealing structural differences reflecting their different oxidation state formulations. The anion component of **5** resides on a crystallographic 3-fold rotation axis and therefore the U–N–U and N_nitride_–U–N_amine_ angles are rigorously 180°, however in **6** the U–N–U angle is bent at 161.2(2)°. In **5** the U1/2–N nitride, amide, and amine distances are 2.0648(2), 2.343(3), and 2.733(5) Å, respectively; these distances reflect the bridging nature of the nitride, that is consistent with other UNU distances,^10^ the anionic, charge rich nature of this component, since the amide distances are longer than usual for Tren-U^IV^ complexes, and possibly a strong *trans*-influence from the nitride since the amine distances are quite long like in related ThPTh and ThAsTh complexes,16c,d,e but in contrast to the situation found in a related terminal Tren-uranium(vi)-nitride.11*b* In **6** the U–N_nitride_ distances are now inequivalent at 2.081(5) and 2.136(5) Å, and the U–N_amide_ and U–N_amine_ distances (av. 2.287(5) and 2.649(5) Å) are now shorter than in **5**, presumably reflecting the neutral formulation of **6** and a reduced nitride *trans*-influence since the N_nitride_–U–N_amine_ angles are now ∼159°.

### Thorium-azide reductions and characterisation of imido products

The reduction of **4**, in contrast to **3**, gives two distinct products, in addition to N_2_ and KN_3_, that are exclusive to the solvent media, Scheme 2.^18^ When aromatic solvents (benzene, toluene) are used instead of securing [{Th(Tren^DMBS^)}_2_(μ-N)][K] the parent imido [{Th(Tren^DMBS^)}_2_(μ-NH)] (**7**) is isolated in 52% crystalline yield. When ethereal solvents (THF, DME) are used the cyclometallated tuck-in-tuck-over,^27^ parent imido [{Th(Tren^DMBS^)}{Th(N[CH_2_CH_2_NSiMe_2_Bu^*t*^]_2_CH_2_CH_2_NSi[μ-CH_2_]MeBu^*t*^)}(μ-NH)][K(DME)_4_] (**8**) is isolated in 46% crystalline yield. The ^1^H NMR spectrum of **7** reveals a resonance at 5.55 ppm that corresponds to one N–H proton; this resonance disappears when the reaction is conducted in D_8_-toluene, suggesting the source of H is aromatic solvent with K–C_6_H_5_/–CH_2_Ph as by-products. In-line with this, **7** does not react with benzyl potassium. The presence of the N–H group is confirmed by a broad absorption at 3390 cm^–1^ in the ATR-IR spectrum of **7**. The ^1^H NMR spectrum of **8** is now more complicated due to the desymmetrisation of one of the Tren^DMBS^ ligands, but the N–H proton resonance can be observed at 5.39 ppm.

The molecular structures of **7** and **8** were determined, Fig. 3b and c. In **7** the Th–N_imido_–Th angle is 145.96(19)° and the imido adopts a trigonal planar geometry in contrast to ThP(H)Th and ThAs(H)Th linkages16c,d that are T-shaped reflecting a sp^2^-NH dianion but p-orbital-dominated bonding of PH and AsH dianions. The Th–N_imido_ distances of 2.331(4) and 2.312(4) Å are similar to the Th–N_amide_ distances (∼2.330 Å) and ∼0.3 Å longer than Th=NR terminal imido bonds.^13^ In **8** the Th–N_imido_–Th angle is 120.9(7)°, reflecting the presence of the tuck-in-tuck-over cyclometallate enforcing a constrained C–Th–N–Th four-membered ring. Despite this, the Th–N_imido_ distances of 2.309(15) and 2.264(15) Å are essentially the same as those in **7**. The Th–C distances of 2.88(2) and 2.78(2) Å are long, as observed in other Th–Tren^DMBS^ cyclometallates.^28^

### Discussion of the contrasting nature of uranium- and thorium-nitride reactivities

The formation and isolation of **5** and **6**, especially the latter under photolytic conditions, is significant because photolysis of terminal uranium(vi)-nitrides10i,11b and a uranium(iv)-nitride generated transiently by reduction6*b* have all resulted in C–H activation of ancillary ligands to produce alkyl-amides or a parent imido-alkyl, respectively. However, **5** and **6** contain quite robust, redox inter-convertible UNU cores, and when N_2_ is eliminated from **3** the nitride secures stabilisation by two uranium Lewis acid centres rather than instigating C–H activation reactions.

Although reduction of **4** does not lead to the isolation of dithorium-nitrides, the isolation of **7** and **8** is instructive. Like **5** and **6**, reduction of an azide precursor, **4**, results in the formation of a ThNTh unit, except in both cases this is protonated. For **7**, the potassium from reduction has been exchanged for a proton suggesting that a transient nitride [{Th(Tren^DMBS^)}_2_(μ-N)]^–^ (**9^–^**) has C–H activated arene solvents, and we note the yield of **7** does not vary when changing the solvent from benzene to toluene. Likewise, in the formation of **8** a μ-NH unit forms again, but this time in the absence of deprotonatable arene solvent, and with the potassium cation sequestered by ethereal solvent, the transient nitride has C–H activated the Tren^DMBS^ to form a tuck-in-tuck-over cyclometallate^27^,^28^ liberating the requisite proton to form the imido group. We deduce that the transient ThNTh unit is highly reactive, certainly as reactive as Th–CR_3_ species that also cyclometallate Tren-ligands due to proximity and entropy effects,^28^ and more reactive than the stable UNU units in **5** and **6**. Indeed, DFT calculations^18^ on [{U(Tren^DMBS^)}_2_(μ-N)]^–^ (**5^–^**) and **9^–^** suggest that the latter contains more polar and ionic metal-nitride linkages, but importantly the frontier molecular orbitals that principally comprise the ThNTh bonding interactions in **9^–^** are destabilised by ∼1.1 (σ) and ∼0.4 (π) eV compared to the corresponding UNU orbitals of **5^–^**. This results in a more basic, effectively superbasic, nucleophilic nitride in **9^–^**, as experimentally inferred by the isolation of **5***versus***7** and **8**, and shown computationally where the anion of **8** is found to be 19.8 kcal mol^–1^ more stable than **9^–^** and formed *via* a transition state with an experimentally accessible barrier of 16.4 kcal mol^–1^.^18^

**Fig. 6:**
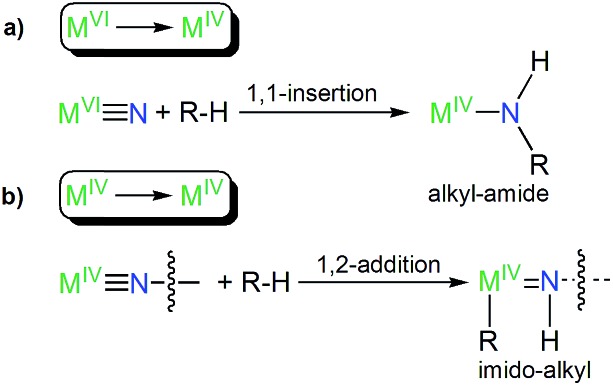
Systematic types of emerging reactivity patterns of actinide-nitrides suggested by this work and ref. 6*b*, 10*i* and 11*b*. (a) C–H activation of a R–H bond by a metal(vi)-nitride produces a metal(iv)-alkyl-amide by 1,1-insertion. (b) C–H activation of a R–H bond by a terminal or bridging metal(iv)-nitride produces a bridging or terminal metal(iv)-imido-alkyl by 1,2-addition; the R group can be coordinated to the metal(iv) ion or be eliminated with a group 1 metal.

The formation of **8** parallels the reactivity of a transiently formed uranium(iv)-nitride that undergoes ligand C–H activation to give a cyclometallated (alkyl) ligand and a uranium(iv) parent imido functionality by 1,2-addition,6*b* since the alternative of 1,1-insertion to give an alkyl-amide as observed for uranium(vi)-nitrides10i,11b would, formally, implicate the likely very unfavourable formation of sub-valent thorium ions. This suggests that there may be a systematic pattern of C–H activation reactions for M^VI^*vs.* M^IV^ ions in this context generally, Fig. 6.

**Fig. 7:**
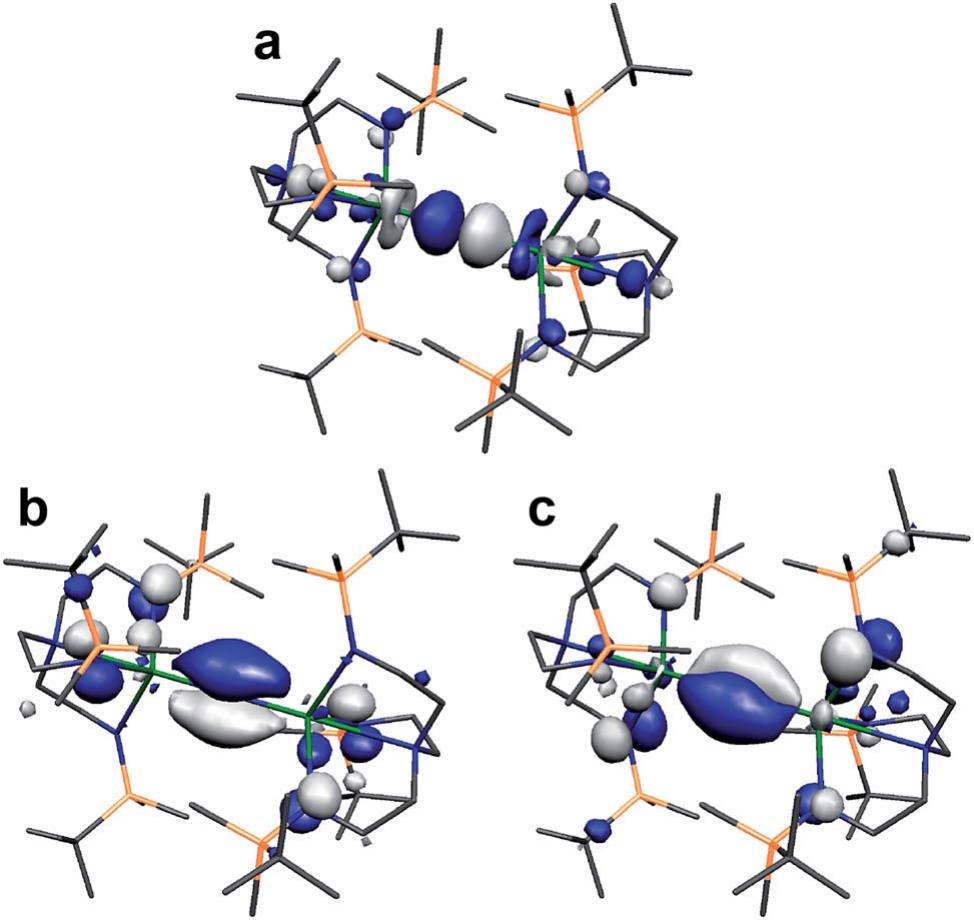
Kohn Sham molecular orbital representations of the principal frontier orbitals of **9^–^** with hydrogen atoms omitted for clarity. (a) HOMO (365a, –1.884 eV); (b) HOMO–7 (358a, –2.598 eV); (c) HOMO–8 (357a, –2.599 eV). The computed thorium-nitride distances are 2.1373 and 2.1373 Å and the computed Th–N_nitride_–Th angle is 180°.

Lastly, we note that the σ-bonding component of the U^IV^NU^IV^ and Th^IV^NTh^IV^ (**9^–^** is depicted in Fig. 7) bonds reported here are higher in energy than the two π-contributions.^18^ This scenario is usually only observed in terminal uranium nitrides and uranyl with short UN and UO bonds.^9^,^11^ Why this is the case here, and whether this is related to the formation of **7** and **8**, is currently unclear and work is on-going to rationalise this observation.

## Conclusions

In conclusion, the synthesis of two uranium- and thorium-azide complexes has provided rare examples of actinide molecular square and triangle complexes. We have prepared two diuranium-nitride complexes in different charge states; these UNU complexes are quite robust, and do not engage in C–H activation chemistry, even under photolytic conditions, unlike terminal uranium(iv/vi)-nitrides. Attempts to prepare a dithorium-nitride complex resulted in the isolation of two parent imido complexes, in-line with the paucity of isolable molecular thorium-nitrides to date. However, the two dithorium-imido products suggest for the first time that reduction of thorium-azides can generate nitrides, and provides evidence that a transient and highly reactive dithorium-nitride is formed, but that this linkage is highly basic and nucleophilic so is capable of activation C–H bonds of arene solvent or the supporting Tren^DMBS^ ligand. The contrasting stabilities of UNU and putative ThNTh units reported here may be related to the general tendency of uranium to engage in more covalent bonding than thorium, on a like-for-like basis. The results here suggest a general pattern of actinide-nitride reactivity where metal(iv)-nitrides, bridging or terminal, activate C–H bonds to produce imido-alkyl combinations, whereas metal(vi)-nitrides produce alkyl-amide linkages, which can be related to the range of accessible oxidation states of these ions during reactions. Lastly, computational studies surprisingly reveal a σ > π energy ordering for the bridging nitride linkages in this study, a phenomenon so far only found in terminal uranium-nitrides and uranyl complexes with very short U–N/–O distances.

## Conflicts of interest

There are no conflicts to declare.

## Supplementary Material

Supplementary information

Crystal structure data
