# Supplementary material for: Thorium- and uranium-azide reductions: a transient dithorium-nitride versus isolable diuranium-nitrides
Source: Chem Sci. 2019 Feb 23;10(13):3738–45. doi: 10.1039/c8sc05473h (PMC6446963; doi:10.1039/c8sc05473h)
Supplement: Supplementary file 1 [file SC-010-C8SC05473H-s001.pdf]

Electronic Supplementary Material (ESI) for Chemical Science.
This journal is © The Royal Society of Chemistry 2019

- Electronic Supplementary Information -

Thorium- and Uranium-Azide Reductions: A Transient Dithorium-Nitride *Versus* Isolable Diuranium-Nitrides

Jingzhen Du,[a,†] David M. King,[b,†] Lucile Chatelain,[a] Erli Lu,[a] Floriana Tuna,[a] Eric J. L. McInnes,[a] Ashley J. Wooles,[a] Laurent Maron,[c,]* and Stephen T. Liddle[a,]*

[a] School of Chemistry, The University of Manchester, Oxford Road, Manchester, M13 9PL, UK.

[b] School of Chemistry, The University of Nottingham, University Park, Nottingham, NG7 2RD, UK.

[c] LPCNO, CNRS & INSA, Université Paul Sabatier, 135 Avenue de Rangueil, Toulouse 31077, France.

*E-mail: steve.liddle@manchester.ac.uk; laurent.maron@irsamc.ups-tlse.fr

Experimental

General

All manipulations were carried out under an inert atmosphere of dry nitrogen using Schlenk techniques, or an MBraun UniLab glovebox. All glassware was dried either by overnight storage in an oven at 150 °C or by flame-drying with subsequent cooling under 10^{-3} mm Hg vacuum followed by repeated alternate evacuation and purging with nitrogen. Oxygen-free nitrogen gas cylinders were supplied by BOC Gases UK. The solvents THF, diethyl ether, toluene and pentane were dried by passage through activated alumina towers and degassed before use. 1,2-dimethoxyethane (DME), hexanes and benzene were distilled from potassium. All solvents were stored over potassium mirrors except for ethers, which were stored over activated 4 Å sieves. Deuterated NMR solvents (d_6-benzene, d_8-toluene, d_8-tetrahydrofuran) were purchased from Goss Scientific Ltd and were distilled from potassium, degassed by three freeze-pump-thaw cycles and stored under nitrogen prior to use. KN_3, NaN_3, and $AgBPh_4$ were dried under vacuum for 6 hours prior to use. UCl_4,[1] $[Th(Cl)_4(THF)_{3.5}]$,[2] $[U(Tren^{DMBS})(I)]$,[3] $[Th(Tren^{DMBS})(I)]$,[4] and KC_8[5] were prepared using literature methods.

^1H, $^{13}C\{^1H\}$, $^{29}Si\{^1H\}$ NMR spectra were recorded on a Bruker 400 spectrometer operating at 400.1, 100.6, and 79.5 MHz, respectively; chemical shifts are quoted in ppm and are relative to TMS (^1H, ^{13}C ^{29}Si). FTIR spectra were recorded on a Bruker Alpha spectrometer with a Platinum-ATR module in the glovebox. UV/Vis/NIR spectra were recorded on a Perkin Elmer L M D™ 750 spectrometer. Data were collected in a 1mm path-length cuvette loaded in an MBraun glovebox and were run versus the appropriate solvent. EPR spectra were measured using X- (9.39 GHz) and S-bands (ca. 3.8 GHz, 5 K) on a Bruker Elexsys E500 spectrometer with an ER4118SMS5 resonator at 5 K; K- (24.0 GHz) and Q-bands (34.0 GHz) were also investigated to confirm the spectral features, but since not all g-values are

observable in those frequency windows they are not included here. Static variable-temperature magnetic moment data were recorded in an applied dc field of 0.1 T on a Quantum Design MPMS XL7 superconducting quantum interference device (SQUID) magnetometer using doubly recrystallised powdered samples. Care was taken to ensure complete thermalisation of the sample before each data point was measured and samples were immobilised in an eicosane matrix to prevent sample reorientation during measurements. Diamagnetic corrections were applied for using tabulated Pascal constants and measurements were corrected for the effect of the blank sample holders (flame sealed Wilmad NMR tube and straw) and eicosane matrix. Crystals were examined variously on a Bruker APEX CCD area detector diffractometer using graphite-monochromated Mo Kα radiation (λ = 0.71073 Å), or on an Oxford Diffraction SuperNova Atlas CCD diffractometer using mirror-monochromated Cu Kα radiation (λ = 1.5418 Å). Cyclic voltammetry experiments were performed using a Metrohm Autolab UK Ltd. PGSTAT20 potentiostat with a three-electrode arrangement in a single compartment cell under N_2, Pt wire working and secondary electrodes and Ag/AgCl reference electrode. Sample solutions were 5 mM in concentration of complex with 0.1 M dry [$^{n}Bu_4N$][BAr^F_4] supporting electrolyte in dry THF, loaded into the cell in the glovebox. CHN microanalyses were carried out by Martin Jennings and Anne Davies (University of Manchester); low C values were often observed, likely the result of incomplete combustion and SiC formation.[6]

Preparation of [{U(Tren^{DMBS})(μ-N_3)}$_4$] (3)

THF (20 ml) was added slowly to a stirring mixture of [U(Tren^{DMBS})(I)] (4.00 mmol, 3.40 g) and NaN_3 (4.20 mmol, 0.27 g) at –78 °C. The mixture was allowed to warm to room temperature with stirring over 16 h. Volatiles were removed *in vacuo* and the product was extracted into hexanes (20 ml). The mixture was filtered to remove the NaI precipitate,

concentrated and stored at –78 °C to yield yellow crystals of **3**. Yield: 0.32 g, 35%. Anal. Calc. for $C_{96}H_{228}N_{28}Si_{12}U_4.C_6H_{14}$: C, 38.89; H, 7.74; N, 12.45. Found: C, 39.09; H, 7.73; N, 12.16. ^{1}H NMR (400 MHz, C_6D_6, 298 K): δ (ppm) 12.33 (bs, 24H, CH_2), 5.24 (s, 108H, C(CH_3)$_3$), 1.33 (s, 72H, Si(CH_3)$_2$) –38.68 (bs, 24H, CH_2). FTIR *v*/cm^{-1}: 2956 (vs), 2924 (vs), 2854 (vs), 2131 (vs, N_3^-), 2096 (s), 1462 (s), 1404 (m), 1378 (m), 1260 (m), 1094 (m), 1021 (m), 900 (w), 802 (m), 722 (w). μ_{eff} (Evans method, C_6D_6 solution, 298 K): 4.28 μ_B. If [{U(Tren^{DMBS})}$_4$(μ:η^1:η^1-N$_3$)$_4$] is extracted into pentane instead of hexane, solvent can be removed from the lattice by drying under vacuum for 4 hours.

Preparation of [{Th(Tren^{DMBS})(μ-N$_3$)}$_3$] (4)

THF (50 ml, –78 °C) was added to pre-cooled (–78 °C) solid [Th(Tren^{DMBS})(I)] (8.45 g, 10.00 mmol) and KN_3 (1.60 g, 20.00 mmol). The resulting mixture was stirred at –78 °C for 1 hour and then left to warm to room temperature and stirred for an additional 24 hours. All volatiles were subsequently removed *in vacuo*. The remaining solids were then extracted in pentane (60 ml) and filtered through celite® to remove the KI precipitate and excess KN_3. All volatiles were removed *in vacuo*, and the residue was washed with HMDSO (2 x 10 mL) and dried *in vacuo* to yield **3** as an analytically pure colourless solid. Yield: 6.54 g, 86%. Colourless crystals were obtained from slow evaporation of a saturated pentane solution at room temperature. Anal. Calcd for $C_{72}H_{171}N_{21}Si_9Th_3$: C, 37.93; H, 7.56; N, 12.90. Found: C, 37.62; H, 7.60; N, 12.75. ^{1}H NMR (400 MHz, C_6D_6, 298 K): δ (ppm) 0.49 (s, 18H, Si(C(CH$_3$)$_3$)(CH_3)$_2$), 1.16 (s, 27H, Si(C(CH_3)$_3$)(CH$_3$)$_2$), 2.49 (t, $^3J_{HH}$ = 5.0 Hz, 6H, CH$_2$CH_2) 3.53 (t, $^3J_{HH}$ = 5.0 Hz, 6H, CH_2CH$_2$). ^{13}C{^{1}H} NMR (101 MHz, C_6D_6, 298 K): δ (ppm) −4.04 (Si(C(CH$_3$)$_3$)(*C*H$_3$)$_2$), 20.22 (Si(*C*(CH$_3$)$_3$)(CH$_3$)$_2$), 27.63 (Si(C(*C*H$_3$)$_3$)(CH$_3$)$_2$), 47.05 (CH$_2$*C*H$_2$), 62.59(*C*H$_2$CH$_2$). ^{29}Si{^{1}H} NMR (79 MHz, C_6D_6, 298 K): δ (ppm) −2.11. FTIR *v*/cm^{-1}: 2950 (s), 2925 (s), 2881(w), 2850 (s), 2131 (vs, N_3^-), 1462 (s), 1401 (w), 1245 (s),

1078 (w), 1058 (w), 926 (vs), 823 (vs), 802 (vs), 785 (s), 770 (s), 714 (s), 657 (s), 546 (s), 631 (w), 446(w).

Preparation of [{U(Tren^{DMBS})}$_2$(μ-N)][K(THF)$_6$] (5) and [{U(Tren^{DMBS})}$_2$(μ-N)] (6)

THF (20 ml) was added slowly to a stirring mixture of **3** (2.568 g, 0.8 mmol) and KC_8 (0.540 g, 4.0 mmol) at –78 °C. The mixture was allowed to warm to room temperature with stirring over 16 h and filtered away from the graphite. Volatiles were removed *in vacuo* and the product was extracted into pentane (60 ml). The solution was filtered, concentrated to 10 ml and stored at –30 °C to yield a 66:34% mixture of **5**:**6** as a dark brown solid. The solid was isolated by filtration, washed with pentane (2 x 2 ml) and dried. Yield: 1.187 g, 41.8% (based on uranium; **5**, 27.6%; **6**, 14.2%). Repetition of this procedure, in DME, Toluene, THF, or Benzene using various ratios of KC_8 (3 to 5 equivalents) always yielded mixtures of the two different bridging nitride species, in various ratios ranging from 77:23 to 50:50% **5**:**6**.

Preparation of [{U(Tren^{DMBS})}$_2$(μ-N)][K(THF)$_6$] (5)

THF (10 ml) was added slowly to a stirring mixture of 77:23% **5**:**6** (1.747 g) and KC_8 (0.074 g, 0.5 mmol) at –78 °C. The mixture was allowed to warm to room temperature with stirring over 16 h and filtered away from the graphite. Volatiles were removed *in vacuo* and the product was extracted into pentane (40 ml). The solution was filtered, concentrated to 5 ml and stored at –30 °C to yield **5** as dark red crystals. The crystals were isolated by filtration, washed with pentane (2 x 2 ml) and dried. Upon prolonged drying loss of 3 molecules of THF occurs. Yield: 1.173 g, 63%. Anal. Calc. for $C_{60}H_{138}N_9KO_3Si_6U_2$: C, 41.96; H, 8.10; N, 7.34. Found: C, 41.45; H, 8.30; N, 7.48. ^{1}H NMR (400 MHz, C_6D_6, 298 K): δ (ppm) 95.53 (s, 12H, CH_2), 15.92 (s, 12H, CH_2), 3.65 (s, 24H, OCH_2, OCH$_2$CH_2), –14.47 (s, 54H, Si(CH_3)$_2$), –33.56 (s, 36H, C(CH_3)$_3$). FTIR ν/cm^{-1}: 2955 (vs), 2924 (vs), 2854 (vs), 1582 (w), 1462 (s),

1377 (s), 1260 (s), 1246 (m), 1072 (s), 1026 (m), 936 (w), 897 (w), 806 (s), 767 (m), 723 (m), 670(w), 650 (w). μ_{eff} (Evans method, C_6D_6 solution, 298 K): 4.02 μ_B.

Preparation of [{U(Tren^{DMBS})}$_2$(μ-N)] (6)

Method 1: Toluene (20 ml) was added slowly to a stirring mixture of 50:50 **5**:**6** (2.790 g) and $AgBPh_4$ (0.65 g, 1.5 mmol) at –78 °C. The mixture was allowed to warm to room temperature with stirring over 16 h and filtered. Volatiles were removed *in vacuo* and the product was extracted into pentane (60 ml). The solution was filtered, concentrated to ~5 ml and stored at –30 °C to yield **6** as dark brown crystals. Yield: 1.388 g, 57%. *Method 2:* Toluene (20 ml) was added slowly to a stirring mixture of **5** (0.86 g, 0.5 mmol) and $AgBPh_4$ (0.43 g, 1.00 mmol) at –78 °C. The mixture was allowed to warm to room temperature with stirring over 16 h and filtered. Volatiles were removed *in vacuo* and the product was extracted into hexanes (10 ml). The solution was filtered, concentrated to ~2 ml and stored at –30 °C to yield **6** as dark brown crystals. Yield: 0.21 g, 30%. *Method 3:* **3** (2.14 g, 0.70 mmol) in toluene (30 ml) was irradiated with a 125 W Hg-lamp in a submersion flask for 7 hours with stirring. The solution was filtered and volatiles were removed *in vacuo*. Extraction into hexanes, concentration, and storage at 5 °C yielded **6** as dark brown crystals. Yield: 0.92 g, 45%. Anal. Calc. for $C_{48}H_{114}N_9Si_6U_2$: C, 39.43; H, 7.86; N, 8.62. Found: C, 39.36; H, 7.87; N, 8.36. ^{1}H NMR (400 MHz, C_6D_6, 298 K): δ (ppm) 24.86 (s, 12H, CH_2), 8.76 (s, 12H, CH_2), –1.19 (s, 54H, Si(CH_3)$_2$), –13.31 (s, 36H, C(CH_3)$_3$). ^{29}Si NMR (C_6D_6, 298 K): δ –104.01. FTIR v/cm^{-1}: 2955 (vs), 2924 (vs), 2854 (vs), 1599 (w), 1462 (s), 1403 (m), 1377 (s), 1260 (s), 1089 (s), 1021 (m), 926 (w), 801 (s), 722 (m), 669 (w). μ_{eff} (Evans method, C_6D_6 solution, 298 K): 3.45 μ_B.

Preparation of [{Th(Tren^{DMBS})}$_2$(μ-NH)] (7)

Benzene (40 ml) was added slowly to a pre-cooled (–78 °C) mixture of **4** (0.76 g, 0.33 mmol) and KC_8 (0.13 g, 1.00 mmol), the mixture was frozen at –78 °C. After addition, the cold bath was removed and the mixture was thawed and warmed up to room temperature and kept stirring for 24 hours, during which the reaction turned into black suspension. After this time, the mixture was filtered, and a colourless filtrate was obtained. Volatiles were removed *in vacuo* and the off-white product was extracted into pentane (10 ml). The solution was filtered, concentrated to 5 mL and stored at -30 °C, yielding **7** as colourless crystals. Yield: 0.20 g, 52%. The same product could be also obtained by using toluene as the solvent. Anal. Calcd for $C_{48}H_{115}N_9Si_6Th_2$: C, 39.73; H, 7.99; N, 8.69. Found: C, 39.76; H, 8.23; N, 8.67. ^1H NMR (400 MHz, C_6D_6, 298 K): δ (ppm) 0.52 (s, 36H, Si(C(CH_3)$_3$)(C*H*$_3$)$_2$), 1.13 (s, 54H, Si(C(C*H*$_3$)$_3$)(CH_3)$_2$), 2.50 (t, $^3J_{HH}$ = 5.0 Hz, 12H, CH_2C*H*$_2$) 3.36 (t, $^3J_{HH}$ = 5.0 Hz, 12H, C*H*$_2$$CH_2$), 5.55 (s, 1H, N*H*). $^{13}C\{^1H\}$ NMR (101 MHz, C_6D_6, 298 K): δ (ppm) −3.17 (Si(C(CH_3)$_3$)(*C*H$_3$)$_2$), 20.64 (Si(*C*(CH_3)$_3$)(CH_3)$_2$), 27.92 (Si(C(*C*H$_3$)$_3$)(CH_3)$_2$), 46.28 (CH_2*C*H$_2$), 64.20 (*C*H$_2$$CH_2$). $^{29}Si\{^1H\}$ NMR (79 MHz, C_6D_6, 298 K): δ (ppm) −1.34. FTIR *v*/cm^{-1}: 3390 (br and w, NH), 2950 (s), 2926 (s), 2851(s), 1462 (s), 1400 (w), 1387 (w), 1358 (w), 1247 (s), 1125 (w), 1110 (w), 1060 (s), 1005 (w), 927 (s), 823 (s), 804 (s), 767 (s), 714 (w), 655 (w), 562 (w), 504(w).

Preparation of [{Th(Tren^{DMBS})}{Th(N[$CH_2CH_2NSiMe_2Bu^t$]$_2$$CH_2CH_2$NSi[μ-$CH_2$]MeBu^t)}(μ-NH)][K(DME)$_4$] (8)

DME (40 ml) was added slowly to a pre-cooled (–78 °C) mixture of **4** (0.76 g, 0.33 mmol) and KC_8 (0.26 g, 2.00 mmol) at –78 °C. After addition, the cold bath was removed and the mixture warmed up to room temperature and kept stirring for 24 hours, during which the reaction turned into black suspension. After this time, the mixture was filtered, and a

colourless filtrate was obtained. Volatiles were removed *in vacuo* and the off-white product was washed by pentane (2 x 10 ml) and then dissolved in DME (5 ml). Evaporation of the pale yellow solution in the glove box at room temperature yielded **8** as colourless crystals. Yield: 0.43 g, 46%. The same product could be also obtained by using THF as the solvent and recrystallized in DME. Anal. Calcd for $C_{64}H_{154}KN_9O_8Si_6Th_2$: C, 41.56; H, 8.39; N, 6.82. Found: C, 40.86; H, 8.42; N, 7.04. ^{1}H NMR (400 MHz, C_6D_6, 298 K): δ (ppm) 0.31-0.48 (m, 33H, $Si(C(CH_3)_3)(C\mathit{H}_3)_2$), 0.96-1.30 (m, 56H, $Si(C(C\mathit{H}_3)_3)(C\mathit{H}_2)$ and Th-$C\mathit{H}_2$), 2.33 (t, $^{3}J_{HH}$ = 4.9 Hz, 12H, $CH_2C\mathit{H}_2$), 2.86 (s, 24H, $OC\mathit{H}_3$), 2.96 (s, 16H, $OC\mathit{H}_2$), 3.20 (t, $^{3}J_{HH}$ = 4.9 Hz, 12H, $C\mathit{H}_2CH_2$), 5.39 (s, 1H, N*H*). $^{13}C\{^{1}H\}$ NMR (101 MHz, C_6D_6, 298 K): δ (ppm) −3.16 ($Si(C(CH_3)_3)(\mathit{C}H_3)_3$), 20.63 ($Si(\mathit{C}(CH_3)_3)(CH_3)_3$), 27.92 ($Si(C(\mathit{C}H_3)_3)(CH_3)_3$), 46.28 ($CH_2\mathit{C}H_2$), 49.30 (Th-$\mathit{C}H_2$), 58.29 ($O\mathit{C}H_3$), 64.62 ($\mathit{C}H_2CH_2$), 71.40 ($O\mathit{C}H_2$). $^{29}Si\{^{1}H\}$ NMR (79 MHz, C_6D_6, 298 K): δ (ppm) −1.33. FTIR *v*/cm^{-1}: 2922 (w), 2876 (w), 2842(m), 1496 (w), 1355 (w), 1238 (w), 1123 (w), 1084 (s), 1032 (w), 927 (s), 807 (s), 763 (s), 723 (s), 649 (w), 553 (w), 503 (s), 483(s). NH stretching absorption was not observed in the ATR-IR spectrum.

Electronic Absorption Spectra

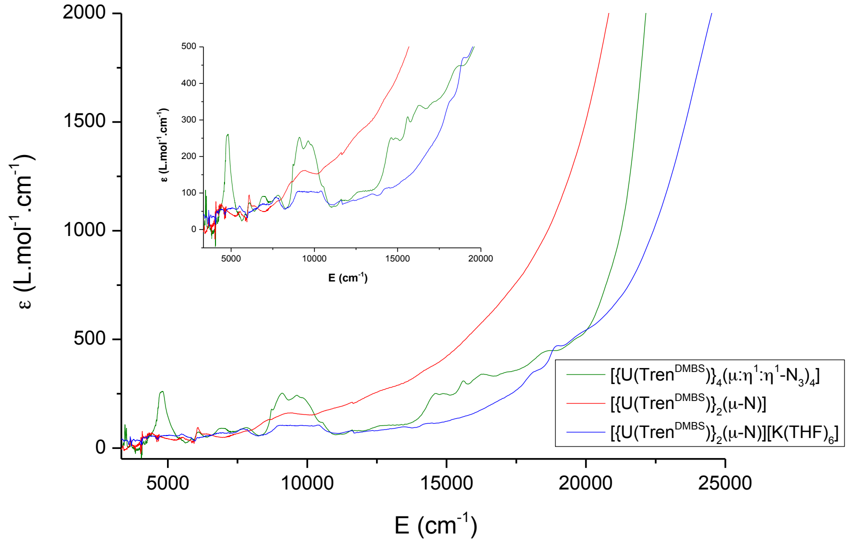

Figure S1. UV-visible-NIR spectra of toluene solutions of **3** (green), **5** (blue), and **6** (red).

Magnetometry

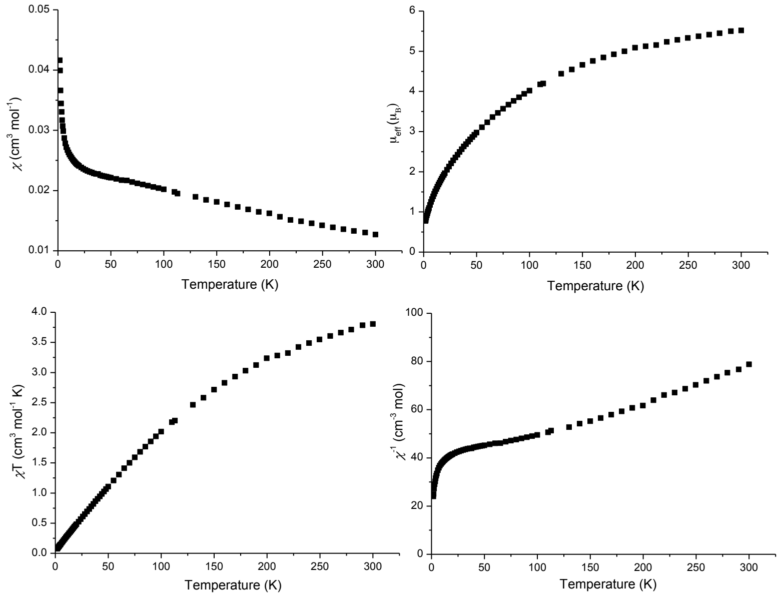

Figure S2. Magnetic susceptibility χ, χT, magnetic moment μ_{eff} and 1/χ data vs T for **3**.

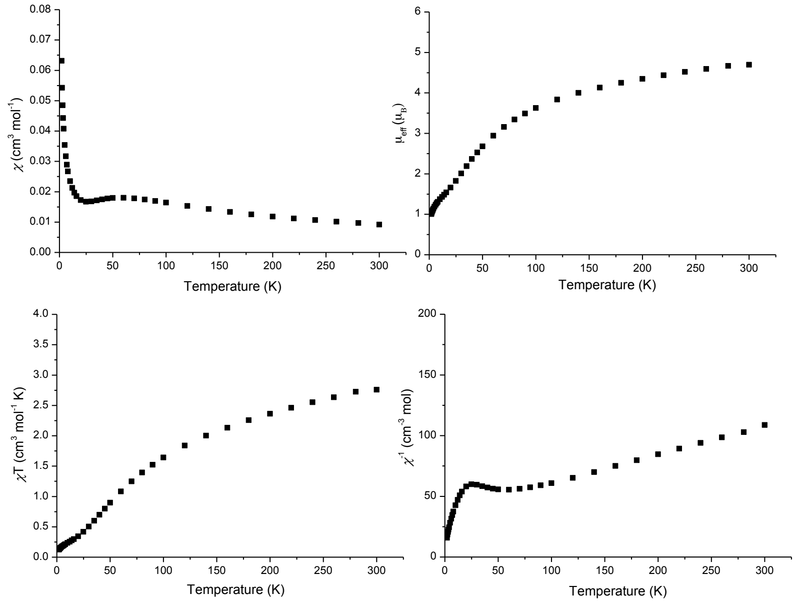

Figure S3. Magnetic susceptibility χ, χT, magnetic moment μ_{eff} and 1/χ data vs T for **5**.

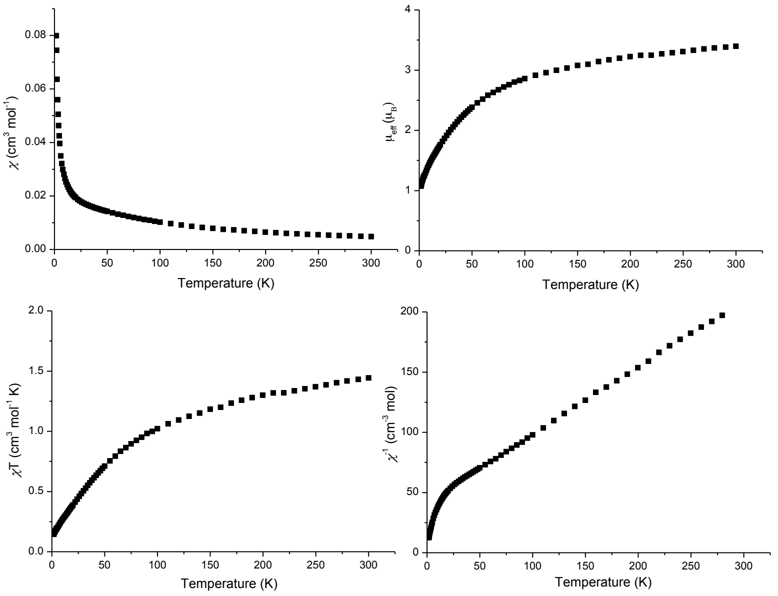

Figure S4. Magnetic susceptibility χ, χT, magnetic moment μ_{eff} and 1/χ data vs T for **6**.

EPR spectroscopy

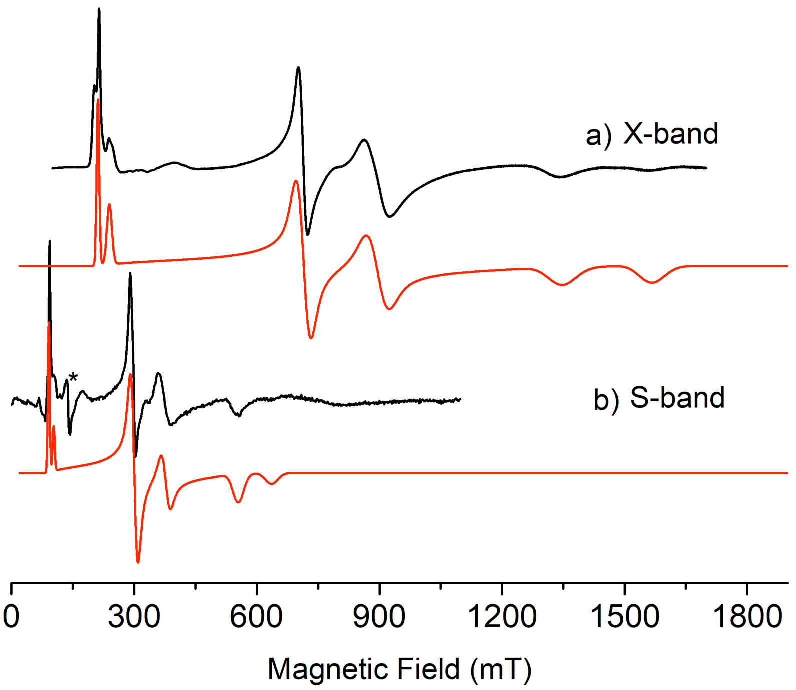

Figure S5. a) X-band (9.39 GHz) and b) S-band (3.87 GHz) EPR spectra of polycrystalline samples of **6** at 5 K. * = cavity signal.

Electrochemistry

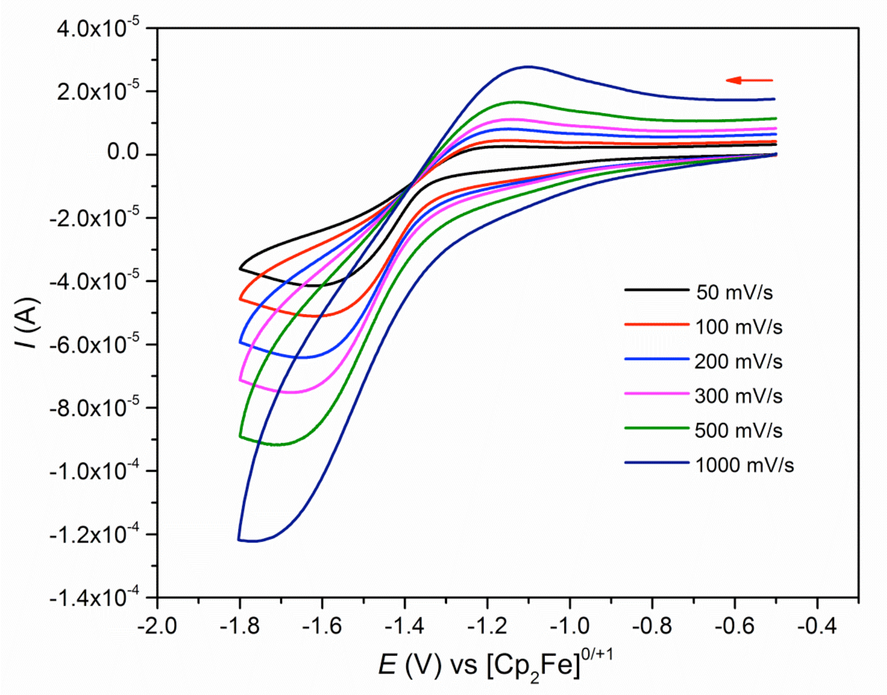

Figure S6. Cyclic voltammogram of 0.1 M **6** in THF at selected sweep rates (0.5 M [N(^{n}Bu)$_4$][BAr$^F{}_4$] supporting electrolyte) *vs.* [Fe(Cp)$_2$]$^{+/0}$ showing a single irreversible redox process. Test solutions of **5** or **6** in THF with [N(^{n}Bu)$_4$][BAr$^F{}_4$] show no evidence of stability issues.

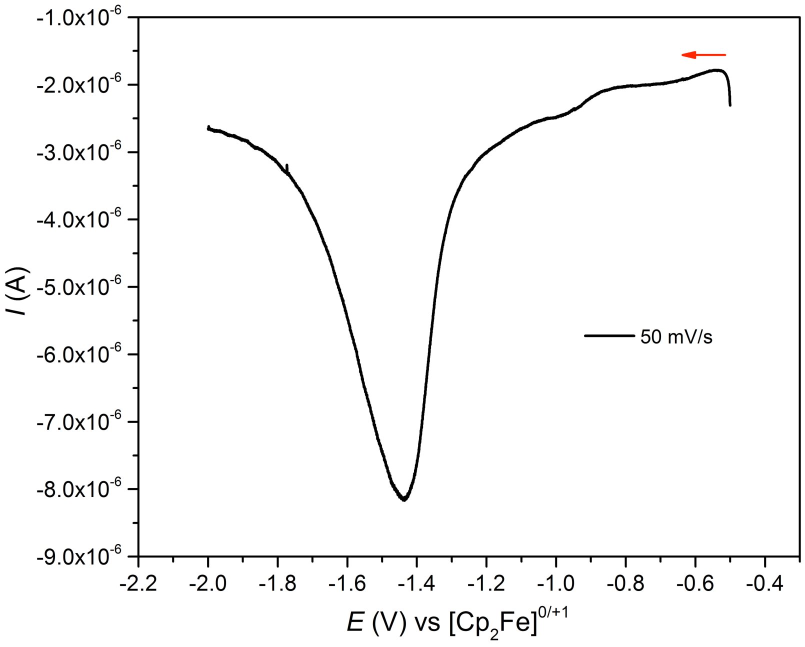

Figure S7. Square wave voltammogram of 0.1 M **6** in THF (0.5 M [N(^{n}Bu)$_4$][BAr$^F{}_4$] supporting electrolyte) *vs.* [Fe(Cp)$_2$]$^{+/0}$.

Computational Details

General

Unrestricted geometry optimisations for [{U(Tren^{DMBS})}$_2$(μ-N)]$^-$ (**5**$^-$) and [{U(Tren^{DMBS})}$_2$(μ-N)] (**6**) were performed using coordinates derived from crystal structures; for [{Th(Tren^{DMBS})}$_2$(μ-N)]$^-$ (**9**$^-$), **5**$^-$ was used with Th substituted for U. No constraints were imposed during the geometry optimisations. Calculations were performed using the Amsterdam Density Functional (ADF) suite version 2012.01.[7,8] The DFT geometry optimisations employed Slater type orbital (STO) triple-ζ-plus polarisation all-electron basis sets (from the ZORA/TZP database of the ADF suite). Scalar relativistic approaches were used within the ZORA Hamiltonian for the inclusion of relativistic effects and the local density approximation (LDA) with the correlation potential due to Vosko *et al.*[9] Gradient corrections were performed using the functionals of Becke and Perdew.[10,11] MOLEKEL[12] was used to prepare the three-dimensional plots of the electron density. The Atoms in Molecules analysis[13,14] was carried out with Xaim-1.0.[15] NBO returned ionic bonding combinations with N-localised lone pairs so these data are not discussed. The enthalpy profile was carried out with the Gaussian 09 suite of programs.[16] Geometries were fully optimised in the gas phase without symmetry constraints, employing the B3PW91 functional.[17,18] The nature of the extrema was verified by analytical frequency calculations. The calculation of electronic energies and enthalpies of the extrema of the potential energy surface (minima and transition states) were performed at the same level of theory as the geometry optimisations. IRC calculations were performed to confirm the connections of the optimised transition states. Th atoms were treated with a small core effective core potential (60MWB), associated with its adapted basis set.[19-21] For the other elements (H, C, O, N and P), Pople's double-ζ basis set 6-31G(d,p) was used.[22,23] This approach follows published methodologies.[24,25]

Kohn Sham Figures

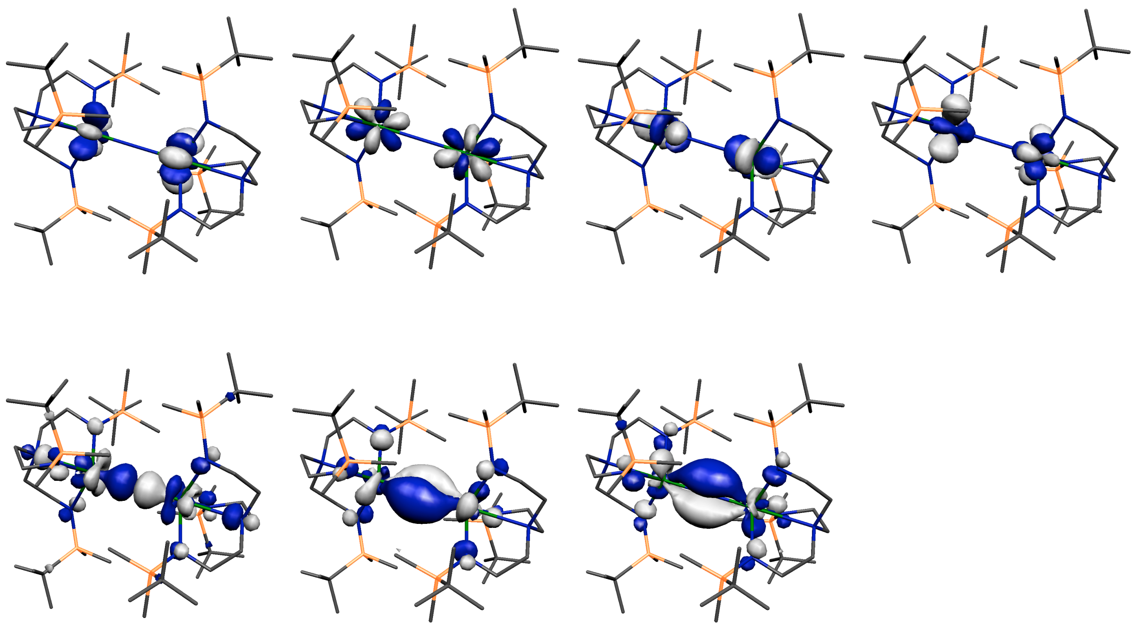

Figure S8. Kohn Sham molecular orbital representations computed by ADF of the principal frontier orbitals of 5^- with hydrogen atoms omitted for clarity. Top left to bottom right: HOMO (369a, 0.004 eV), HOMO−1 (368a, −0.015 eV), HOMO−2 (367a, −0.016 eV), HOMO−3 (366a, −0.053 eV), HOMO−10 (359a, −2.912 eV), HOMO−11 (358a, −2.985 eV), HOMO−12 (357a, −2.985 eV).

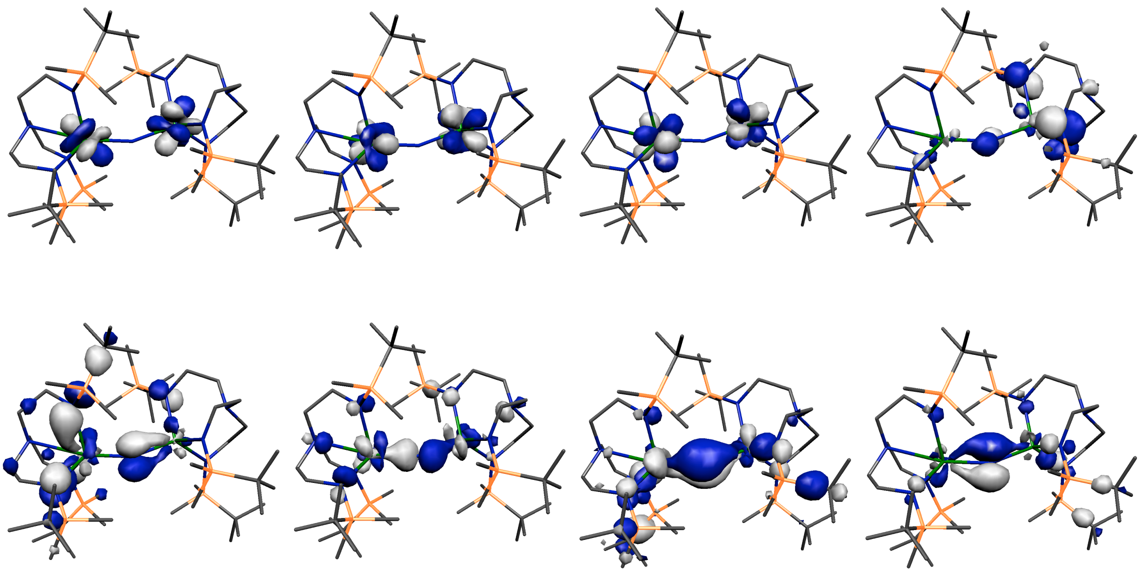

Figure S9. Kohn Sham molecular orbital representations computed by ADF of the principal frontier orbitals of 6 with hydrogen atoms omitted for clarity. Top left to bottom right: HOMO (368a, −2.817 eV), HOMO−1 (367a, −2.854 eV), HOMO−2 (366a, −2.871 eV), HOMO−7 (361a, −4.907 eV), HOMO−8 (360a, −4.984 eV), HOMO−9 (359a, −5.395 eV), HOMO−10 (358a, −5.562 eV), HOMO−11 (357a, −5.582 eV).

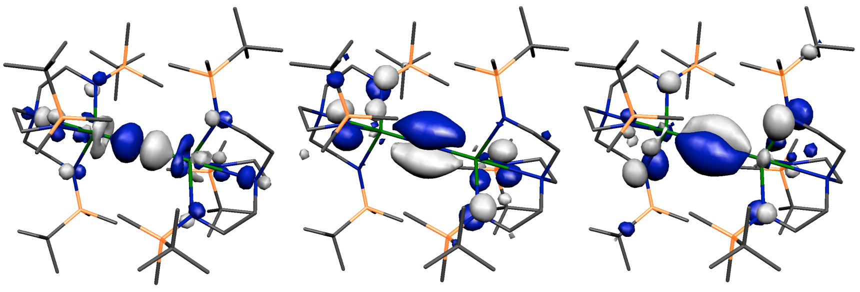

Figure S10. Kohn Sham molecular orbital representations computed by ADF of the principal frontier orbitals of 9^- with hydrogen atoms omitted for clarity. Left to right: HOMO (365a, −1.884 eV), HOMO−7 (358a, −2.598 eV), HOMO−8 (357a, −2.599 eV).

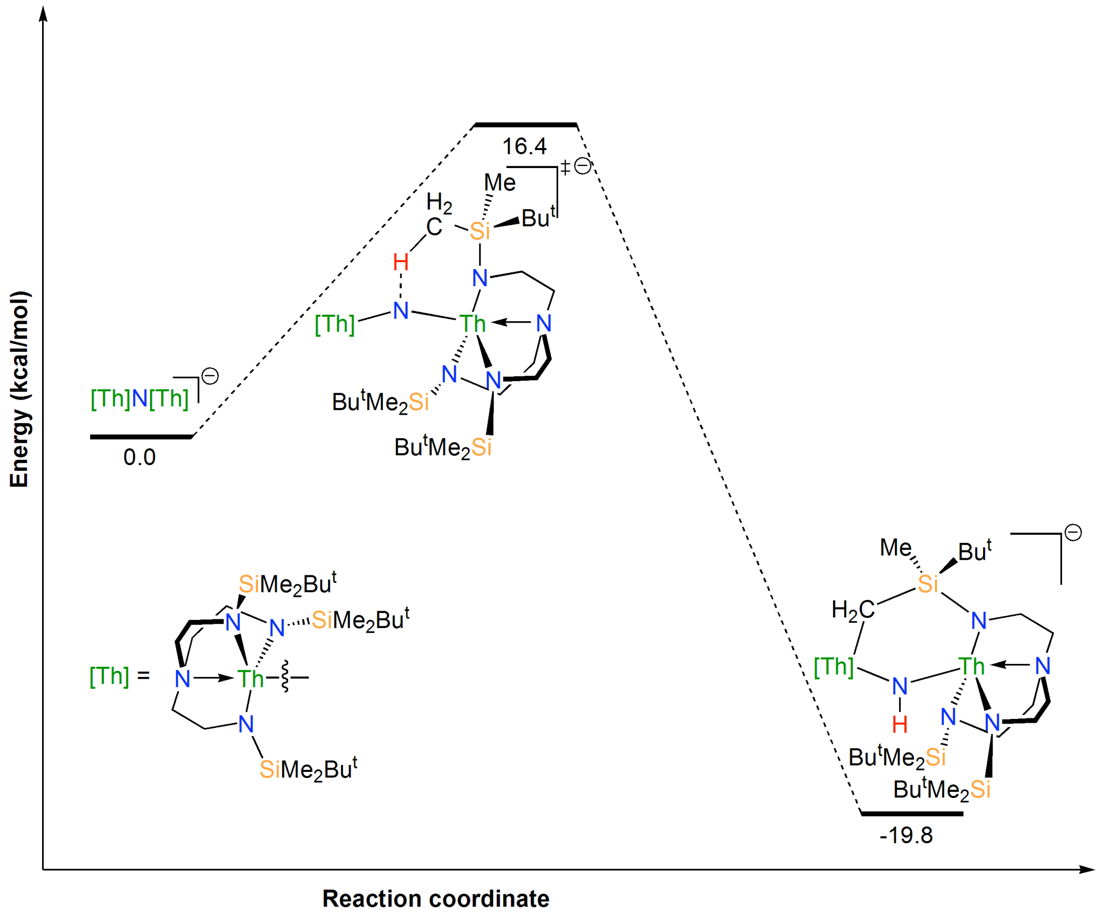

Figure S11. Enthalpy profile computed by Gaussian for the internal proton transfer at 298 K.

Table S1. Selected computed DFT (ADF) and QTAIM data.

	Bond lengths and indices		MDC atomic charges		QTAIM parameters[f]			
**Entry**[a]	**M-N**[b]	**BI**[c]	q_M[d]	Q_N[e]	**ρ(r)**	$\nabla^2\rho(r)$	$H(r)$	$\varepsilon(r)$
**5**$^-$	2.065	1.31	2.32	-1.36	0.15	0.35	-0.08	0.01
	2.065	1.31	2.32		0.15	0.35	-0.08	0.01
**6**	2.090	1.62	2.39	-1.30	0.16	0.33	-0.08	0.22
	2.084	1.65	2.59		0.16	0.33	-0.08	0.29
**9**$^-$	2.138	1.24	2.16	-1.11	0.14	0.29	-0.07	0.01
	2.138	1.24	2.16		0.14	0.29	-0.07	0.01

[a] All molecules geometry optimised without symmetry constraints at the LDA VWN BP TZP/ZORA level. [b] Calculated $M\text{-}N_{nitride}$ distances (Å). [c] Mayer bond indices. [d] MDC-q charges on metals. [e] MDC-q charges on nitrides. [f] QTAIM topological electron density [ρ(r)], Laplacian [$\nabla^2\rho$(r)], electronic energy density [H(r)], and ellipticity [$\varepsilon(r)$] bond critical point data. For reference, the $M\text{-}N_{amide}$ and $M\text{-}N_{amine}$ Mayer bond orders average 0.7 and 0.20, respectively.

Final Coordinates and Energies of Computed Structures

5⁻ (from ADF)

C	1.744779	-3.770466	-5.344140
C	-3.248451	2.419994	-5.421960
C	1.634684	-0.305773	-5.367564
C	4.026013	-2.858005	-4.877087
C	-3.662199	0.199616	-4.324949
C	2.614993	-3.058071	-4.282879
C	-3.771560	1.726879	-4.142954
C	-5.261449	2.096688	-3.975928
C	2.727058	-3.964099	-3.040168
C	-0.001914	-1.809830	-3.236743
C	3.865887	0.103294	-2.967686
C	-0.895296	1.856338	-3.053967
C	2.671171	4.328578	-2.564572
C	-2.820633	4.186042	-2.483175
C	5.015594	-0.350906	-2.076296
C	-2.603274	-4.621939	-1.878257
C	2.940759	4.951877	-1.180167
C	4.455959	5.215830	-1.044240
C	2.208391	6.310991	-1.093303
C	-4.365334	-2.126444	-0.610330
C	-4.418927	2.023872	-0.557750
C	5.221048	1.460773	-0.436833
C	-5.381528	0.870754	-0.293808
C	0.373317	3.691194	-0.030398
C	-0.375233	-3.664172	0.014214
C	5.359835	-0.876036	0.294229
C	-5.240923	-1.464011	0.448264
C	4.396738	-2.029777	0.547625
C	4.345658	2.115916	0.624823
C	-2.188610	-6.308733	1.106430
C	-4.428892	-5.200018	1.103011
C	-2.909336	-4.944716	1.205138
C	2.593496	4.608468	1.897124
C	-5.034460	0.356934	2.080092
C	2.806985	-4.196597	2.465616
C	-2.607682	-4.321431	2.582831
C	-3.882106	-0.087760	2.974688
C	-2.667001	3.991320	3.015666
C	0.899065	-1.858750	3.083052
C	0.002179	1.790220	3.216882
C	5.276985	-2.158738	3.956837
C	3.796623	-1.755654	4.130412
C	-2.591387	3.081836	4.258607
C	3.719658	-0.225843	4.307562
C	-4.016738	2.903412	4.824942
C	-1.733798	3.782519	5.337177

C	3.262856	-2.434423	5.413114
C	-1.639876	0.331003	5.369063
H	-3.840230	2.098756	-6.300347
H	1.684264	-3.192290	-6.279910
H	1.075699	-0.828670	-6.160052
H	2.179518	-4.756654	-5.595527
H	4.018504	-2.189029	-5.752319
H	2.615344	-0.025540	-5.782174
H	-2.196353	2.171233	-5.626640
H	-3.328523	3.517021	-5.357587
H	4.441519	-3.828517	-5.210011
H	-4.291797	-0.139351	-5.169234
H	0.717211	-3.945228	-4.990585
H	1.095472	0.625803	-5.136152
H	-5.825929	1.823832	-4.888312
H	-2.629851	-0.113750	-4.539120
H	4.729876	-2.442359	-4.140149
H	4.132264	-0.159669	-4.006919
H	-5.408293	3.176312	-3.813283
H	-0.557876	2.465705	-3.906966
H	-0.718202	-1.616353	-4.049796
H	-3.987036	-0.340689	-3.423385
H	3.220848	-4.920010	-3.298483
H	-5.728644	1.561326	-3.135735
H	3.065390	4.979032	-3.367919
H	-2.327397	4.678010	-3.337745
H	-0.774727	0.800907	-3.330617
H	-0.101266	-2.869547	-2.963763
H	3.822344	1.208812	-2.957977
H	1.741546	-4.208724	-2.616743
H	-3.861730	4.545514	-2.451845
H	1.596133	4.190316	-2.753383
H	3.147570	3.341865	-2.664122
H	-2.361154	-3.938632	-2.707251
H	5.994534	0.030275	-2.436648
H	3.313661	-3.485736	-2.241812
H	5.052580	-1.448261	-2.096726
H	-0.315636	-1.216572	-2.363591
H	-0.216579	2.048678	-2.212653
H	-1.982897	-5.525643	-1.996664
H	4.786678	5.945644	-1.807979
H	2.540301	6.976730	-1.912894
H	-3.655596	-4.926802	-1.995918
H	-2.322294	4.532416	-1.564216
H	-4.485192	-1.579182	-1.564302
H	5.050016	4.301673	-1.194178
H	5.102691	2.022436	-1.373053
H	-4.933855	2.734064	-1.229127
H	1.116778	6.201790	-1.180869
H	-5.533550	0.328344	-1.236667

H	0.073076	3.997843	-1.041768
H	-4.786215	-3.129122	-0.804529
H	4.719450	5.632766	-0.059217
H	2.419190	6.830764	-0.145349
H	0.076023	-2.946495	-0.683379
H	4.242095	-2.579545	-0.400033
H	6.296118	1.483060	-0.159253
H	6.351413	-1.233255	-0.055691
H	-4.717192	-5.621053	0.126673
H	-6.372163	1.230926	0.055720
H	-2.392011	-6.813037	0.148220
H	0.080384	-4.649378	-0.171387
H	-6.315916	-1.488622	0.170887
H	-0.018587	2.674868	0.132813
H	-4.267412	2.583768	0.384345
H	-0.135405	4.364117	0.676788
H	4.769519	3.115213	0.829646
H	-1.097630	-6.208614	1.208648
H	5.509253	-0.339483	1.240719
H	4.911614	-2.748184	1.210109
H	-0.091162	-3.351232	1.027350
H	-5.014009	-4.281651	1.261404
H	-5.122428	-2.020069	1.387965
H	-4.746124	-5.924193	1.877882
H	4.460410	1.559912	1.574477
H	-2.534937	-6.983394	1.912490
H	2.129693	5.606652	1.947590
H	2.139459	-4.541457	1.660833
H	3.666053	4.729132	2.113693
H	-5.074424	1.454376	2.093088
H	3.822011	-4.549839	2.227017
H	-6.011796	-0.024072	2.444448
H	-3.236279	3.519210	2.201211
H	0.341447	-1.451925	2.224767
H	0.021244	2.534629	2.410386
H	2.160189	3.996868	2.703680
H	-1.527194	-4.199729	2.748611
H	-3.068085	-3.327865	2.688397
H	-1.667987	4.228112	2.621187
H	0.525523	0.899309	2.845584
H	-3.839696	-1.193325	2.977173
H	5.752732	-1.633244	3.115031
H	-2.996025	-4.963681	3.395696
H	-3.160029	4.950546	3.262846
H	2.492661	-4.693033	3.397612
H	0.363967	-2.752792	3.438039
H	5.397296	-3.241195	3.791356
H	4.061860	0.303967	3.406076
H	-4.146168	0.187414	4.011442
H	0.582499	2.196357	4.060054

H	-4.711694	2.496003	4.075302
H	0.850292	-1.111852	3.887371
H	5.852547	-1.900420	4.866469
H	2.694414	0.115240	4.514818
H	-4.423777	3.881491	5.146558
H	4.352569	0.100341	5.154404
H	-0.708568	3.977730	4.988652
H	3.354569	-3.531065	5.365221
H	-1.286437	-0.682518	5.122765
H	-4.037423	2.238172	5.702848
H	-2.181522	4.757957	5.607543
H	2.205654	-2.193844	5.602053
H	-2.603593	0.228232	5.893219
H	-0.922714	0.772219	6.080861
H	3.841076	-2.095288	6.293789
H	-1.667305	3.186213	6.261274
N	2.585345	-0.484504	-2.523954
N	-3.135711	1.531410	-1.099712
N	4.776718	0.071540	-0.679579
N	-2.950566	-2.157533	-0.185712
N	-0.011844	-0.005399	0.002223
N	2.932116	2.155365	0.197492
N	-4.797935	-0.072720	0.684604
N	3.114739	-1.541539	1.096042
N	-2.602437	0.495483	2.522184
Si	1.759368	-1.370025	-3.784750
Si	-2.682552	2.282232	-2.613142
Si	-2.249312	-3.759683	-0.207438
Si	2.251910	3.765699	0.216037
Si	2.683752	-2.294910	2.613523
Si	-1.756109	1.378685	3.772267
U	2.031794	0.030461	-0.289275
U	-2.058713	-0.031270	0.293223

Energy: −971.78905733 eV

6 (from ADF)

H	-3.030341	0.727611	-7.129994
H	-1.441376	1.168006	-6.470629
H	-4.083646	2.779996	-6.336272
C	-2.391268	0.665272	-6.230703
H	-2.175046	-0.398935	-6.054954
H	5.652952	-1.706833	-5.617523
H	-2.550640	3.362419	-5.655658
H	4.038195	-0.966869	-5.625135
H	-5.055271	0.546339	-5.667475
C	-3.454729	2.777535	-5.427478
H	5.482630	0.052478	-5.429839
C	5.034390	-0.914585	-5.158189
C	-3.113240	1.323346	-5.027847
H	3.863363	1.759798	-4.838776

H	0.054283	2.181086	-4.807881
C	-4.418247	0.544601	-4.764508
H	-4.010643	3.314764	-4.646070
H	-4.226572	-0.505745	-4.502202
H	-0.719063	-0.902665	-4.188824
H	5.205672	-3.288872	-3.731135
H	1.857686	-0.524321	-4.429489
H	-0.801468	3.440799	-3.892635
H	5.053578	2.209405	-3.599764
H	-5.010043	0.986278	-3.948858
C	-0.454218	2.396328	-3.855762
C	4.026578	1.841317	-3.752510
H	3.514440	-2.752595	-3.815242
H	7.084266	-1.683832	-3.582419
C	4.977140	-1.115070	-3.625287
C	4.485057	-2.548172	-3.340430
H	3.339126	2.609025	-3.368901
Si	-1.904212	1.221608	-3.502447
H	6.824342	0.034908	-3.225550
H	-3.804563	-2.505828	-3.307519
C	-1.350102	-0.578700	-3.350647
C	6.410156	-0.973589	-3.069332
H	0.296888	2.333531	-3.055069
H	-2.229562	-1.235446	-3.314778
C	1.979520	-0.419969	-3.340361
H	-4.318835	-4.153506	-2.899416
H	-5.443595	-2.796245	-2.698443
H	1.736281	-1.393957	-2.889877
C	-4.390512	-3.098306	-2.590176
Si	3.708631	0.174334	-2.891822
H	-4.337491	2.591760	-2.666118
H	1.221468	0.300930	-3.004071
H	4.379380	-2.729747	-2.261946
H	-0.759103	-0.738906	-2.433632
H	6.458852	-1.203142	-1.994261
H	-3.045536	3.694970	-2.210819
H	2.949248	4.870809	-1.927624
C	-3.528941	2.739492	-1.927110
N	-2.594173	1.607903	-1.925920
H	1.388181	4.040502	-1.789514
H	-5.151556	0.874373	-1.696594
H	-4.394268	-5.795251	-1.107550
H	-6.503626	-4.242511	-1.011717
H	5.801528	0.847438	-1.322918
C	2.341837	4.221747	-1.271734
H	2.866258	3.257754	-1.183305
H	-1.324027	-2.638192	-1.409880
H	2.443431	-3.097456	-1.267104
N	3.698412	0.375218	-1.140722
H	1.867487	6.873121	-0.761340

H	-5.094929	3.544485	-0.638924
H	1.240306	-4.345763	-0.898686
Si	-3.745573	-2.862992	-0.812080
H	-5.938070	-1.237253	-0.871557
H	-1.739301	-4.329157	-1.120600
H	4.619073	-4.004177	-0.666012
H	0.336679	6.006304	-0.525452
C	-5.388815	0.790259	-0.630377
C	-4.166822	2.945043	-0.554714
H	-6.430245	1.136803	-0.472521
C	4.961762	0.941825	-0.615099
C	-1.912213	-3.303799	-0.762011
C	-4.252294	-5.552769	-0.042574
H	0.810111	-2.645110	-0.705038
C	1.631020	-3.369089	-0.578070
C	-6.253633	-4.052275	0.042995
H	-3.189659	-5.702934	0.198993
H	4.863387	2.027034	-0.430823
H	-0.406248	2.736079	-0.517366
C	1.327790	6.188225	-0.082340
H	3.032525	-5.942054	-0.173354
H	-6.682875	-3.078951	0.321408
C	-5.242455	-0.675785	-0.220474
N	-3.854078	-1.173576	-0.324794
H	4.044276	5.942235	0.047295
H	-4.830444	-6.293119	0.539584
H	-3.478979	3.501803	0.097153
H	-6.771450	-4.820975	0.644874
H	5.837641	-4.598773	0.475297
C	2.131092	4.881782	0.103290
C	-4.730875	-4.121143	0.301611
C	4.927605	-3.978548	0.387977
N	-4.432473	1.648735	0.109009
U	-2.137669	0.357884	-0.026476
N	-0.091664	-0.030756	-0.108920
H	5.503151	-0.826920	0.453564
H	-1.066804	4.192930	0.249697
H	-1.494600	-3.237173	0.253522
H	5.207731	-2.942208	0.624875
C	5.385662	0.241981	0.669610
H	4.145717	4.339738	0.802645
C	3.509198	5.231639	0.703237
H	4.346425	-6.577008	0.838962
C	-0.498933	3.270083	0.441503
U	1.933281	0.147926	0.375879
H	1.180186	6.721356	0.869245
C	3.446036	-5.936296	0.847028
H	6.356584	0.631180	1.038555
H	-5.632409	-0.810864	0.806153
Si	1.167068	3.657010	1.258663

C	3.819935	-4.509252	1.317051
H	-1.072014	2.653326	1.159976
H	2.711388	-6.413653	1.512338
Si	2.221232	-3.397698	1.219915
H	3.425518	5.702561	1.695256
H	-5.548423	2.711112	1.593094
C	-4.833283	1.867117	1.519306
H	4.747933	2.429765	1.547770
N	2.023779	2.143430	1.551142
C	-4.466434	-3.860849	1.796751
N	2.416406	-1.701340	1.626241
H	-5.338401	0.961167	1.875944
N	4.329482	0.379448	1.703249
H	-4.783078	-2.852476	2.100296
H	0.731359	-5.267051	2.016404
H	-3.400978	-3.966468	2.049017
H	-3.125667	3.060326	2.110704
H	-5.021291	-4.586654	2.418581
N	-2.637845	0.976342	2.164141
C	4.354839	1.737485	2.302786
H	5.051332	-1.482137	2.391393
H	5.221700	-5.314236	2.787537
C	0.920623	-4.227822	2.327241
C	-3.586958	2.088703	2.367744
H	-0.031560	-3.681813	2.283398
H	-2.150418	-2.158148	2.362536
C	4.374517	-4.605088	2.755710
H	0.066394	5.398020	2.679422
C	2.960897	2.203270	2.695568
C	4.368607	-0.694718	2.737681
C	0.765048	4.570286	2.881530
H	3.065757	3.220332	3.110463
H	4.751972	-3.638638	3.122461
H	-0.564062	-1.365879	2.557419
H	5.043214	1.761179	3.170571
C	2.973566	-1.296555	2.923805
H	3.619319	-4.966743	3.470168
C	-1.520848	-1.581810	3.057071
H	1.648586	5.005633	3.372734
H	1.235765	-4.250974	3.382489
H	-3.911468	2.201991	3.415665
H	0.281822	3.895818	3.604048
H	2.592448	1.590371	3.539124
H	3.029417	-2.122955	3.653517
H	2.327454	-0.539560	3.408704
H	-4.733974	-1.001650	3.545531
H	4.766946	-0.300453	3.690070
Si	-2.422885	-0.016290	3.600147
H	0.184554	1.439095	3.610041
H	-1.306681	-2.237199	3.914629

C	-4.116357	-0.519538	4.318021
H	0.515653	-0.074221	4.476583
H	-1.832407	2.926109	4.466267
H	-4.685649	0.332291	4.721562
C	0.069672	0.926311	4.576657
H	-3.992122	-1.243491	5.138296
C	-1.403597	0.834401	5.016672
H	0.664492	1.477790	5.327096
C	-1.918689	2.254786	5.333052
H	-2.970415	2.254416	5.660283
H	-1.324429	2.699006	6.152490
H	-1.153416	-1.053746	6.138701
C	-1.484647	-0.016289	6.304329
H	-2.504774	-0.049630	6.715308
H	-0.829451	0.414296	7.083634

Energy: – 970.03116789 eV

9⁻ (from ADF)

C	1.751679	-3.812852	-5.359285
C	-3.286565	2.417785	-5.461567
C	1.717321	-0.326024	-5.434461
C	4.053796	-2.915696	-4.953526
C	-3.721465	0.217849	-4.332055
C	2.651869	-3.090778	-4.331306
C	-3.834008	1.748557	-4.180651
C	-5.326069	2.120422	-4.041568
C	2.777287	-3.972497	-3.072371
C	0.079819	-1.760469	-3.257715
C	3.990173	0.071707	-3.004993
C	-0.981592	1.912462	-3.022341
C	2.722375	4.356393	-2.566896
C	-2.936236	4.227080	-2.508439
C	5.135910	-0.369154	-2.092347
C	-2.711396	-4.677433	-1.880413
C	2.980641	5.005634	-1.192062
C	4.489472	5.305091	-1.062565
C	2.212866	6.344962	-1.120515
C	-4.479874	-2.157774	-0.575634
C	-4.543713	2.022597	-0.575980
C	5.338227	1.469879	-0.471260
C	-5.495509	0.858012	-0.293004
C	0.463231	3.675381	0.004846
C	-0.469282	-3.676320	-0.033754
C	5.482040	-0.863747	0.292125
C	-5.349322	-1.472719	0.481197
C	4.529010	-2.028261	0.566934
C	4.468353	2.148968	0.588157
C	-2.211881	-6.345092	1.134184
C	-4.475990	-5.278673	1.122631
C	-2.960922	-4.995751	1.214824

C	2.683785	4.663831	1.894538
C	-5.147353	0.374295	2.094148
C	2.906718	-4.232236	2.496144
C	-2.665066	-4.343495	2.580593
C	-4.000020	-0.059686	3.009298
C	-2.722871	3.991884	3.047927
C	0.977537	-1.893152	3.033021
C	-0.079905	1.742996	3.268798
C	5.325838	-2.188580	4.035072
C	3.844782	-1.776024	4.174199
C	-2.640935	3.108615	4.309630
C	3.770649	-0.242807	4.322386
C	-4.061682	2.952492	4.893563
C	-1.761749	3.822508	5.361123
C	3.277928	-2.429246	5.454918
C	-1.739902	0.342010	5.441417
H	-3.862384	2.083042	-6.345700
H	1.664724	-3.246000	-6.299820
H	1.161408	-0.853462	-6.226455
H	2.175607	-4.803699	-5.611585
H	4.034055	-2.270896	-5.846417
H	2.697724	-0.042638	-5.848003
H	-2.231306	2.162379	-5.641937
H	-3.364032	3.515931	-5.418196
H	4.461445	-3.896376	-5.265883
H	-4.326764	-0.135723	-5.188009
H	0.734257	-3.980165	-4.973918
H	1.173717	0.604730	-5.207872
H	-5.882473	1.821317	-4.950772
H	-2.683918	-0.101360	-4.510942
H	4.769315	-2.481150	-4.239078
H	4.280372	-0.193344	-4.037347
H	-5.474329	3.204182	-3.910521
H	-0.606660	2.562267	-3.828575
H	-0.646893	-1.553048	-4.057811
H	-4.075124	-0.304872	-3.430697
H	3.241815	-4.945053	-3.321956
H	-5.800139	1.609454	-3.189888
H	3.085816	5.010655	-3.381682
H	-2.529286	4.723611	-3.404121
H	-0.846913	0.871661	-3.346682
H	-0.049414	-2.813334	-2.970233
H	3.948410	1.179129	-3.002972
H	1.798036	-4.185050	-2.617899
H	-3.975006	4.571216	-2.383749
H	1.651413	4.177517	-2.744811
H	3.235235	3.387308	-2.658923
H	-2.392018	-4.028523	-2.711045
H	6.115246	0.003012	-2.462415
H	3.397348	-3.490797	-2.301635

H	5.170763	-1.467200	-2.096906
H	-0.194891	-1.145509	-2.384367
H	-0.343285	2.061520	-2.140982
H	-2.162240	-5.628826	-1.968240
H	4.807139	6.024522	-1.841626
H	2.531769	7.014372	-1.942282
H	-3.780206	-4.898773	-2.027901
H	-2.364230	4.585138	-1.638042
H	-4.603229	-1.614994	-1.534045
H	5.100980	4.399174	-1.191014
H	5.215876	2.019086	-1.414772
H	-5.082552	2.723523	-1.238839
H	1.125187	6.204921	-1.215203
H	-5.641255	0.302756	-1.229623
H	0.131464	3.969250	-1.000867
H	-4.924435	-3.152209	-0.761415
H	4.742593	5.748116	-0.086307
H	2.401239	6.877013	-0.174508
H	-0.061630	-2.908448	-0.704973
H	4.376952	-2.585683	-0.378689
H	6.413992	1.504588	-0.195491
H	6.476731	-1.224995	-0.046283
H	-4.757237	-5.728707	0.157210
H	-6.490246	1.220836	0.043953
H	-2.407267	-6.868789	0.184768
H	0.008573	-4.636422	-0.283878
H	-6.425425	-1.510328	0.206810
H	0.113018	2.643997	0.177914
H	-4.395805	2.588712	0.365324
H	-0.055311	4.331592	0.720143
H	4.914265	3.140422	0.783951
H	-1.122771	-6.218978	1.230375
H	5.626896	-0.313814	1.232021
H	5.066068	-2.736105	1.223155
H	-0.162022	-3.409974	0.986659
H	-5.074463	-4.364781	1.256181
H	-5.225698	-2.016624	1.427645
H	-4.783213	-5.987176	1.915966
H	4.585668	1.597508	1.542219
H	-2.539482	-7.015342	1.951949
H	2.221252	5.663006	1.942001
H	2.236575	-4.582370	1.695256
H	3.756491	4.782936	2.112772
H	-5.184457	1.472257	2.092713
H	3.921533	-4.584523	2.254186
H	-6.125768	0.002564	2.467386
H	-3.318713	3.512754	2.256628
H	0.454387	-1.493847	2.148036
H	-0.037184	2.520430	2.494032
H	2.248351	4.055553	2.702923

H	-1.587115	-4.186856	2.735120
H	-3.156308	-3.363523	2.675809
H	-1.727469	4.200907	2.628622
H	0.389817	0.842894	2.849893
H	-3.959986	-1.167259	3.016784
H	5.814499	-1.686836	3.186123
H	-3.026102	-4.984959	3.406569
H	-3.192304	4.965865	3.282545
H	2.596779	-4.726257	3.431183
H	0.430374	-2.785096	3.374053
H	5.442771	-3.275375	3.898189
H	4.146669	0.268806	3.423748
H	-4.290345	0.213895	4.039789
H	0.534603	2.085247	4.116086
H	-4.762414	2.522549	4.161894
H	0.894540	-1.140369	3.829465
H	5.890201	-1.909909	4.945726
H	2.741015	0.106854	4.490626
H	-4.465824	3.939840	5.189410
H	4.377223	0.095717	5.183496
H	-0.740024	4.000298	4.992681
H	3.350653	-3.527960	5.422941
H	-1.308542	-0.644613	5.210244
H	-4.075061	2.312941	5.790482
H	-2.195248	4.807900	5.618676
H	2.221535	-2.168125	5.620703
H	-2.721303	0.169774	5.910696
H	-1.093993	0.822421	6.193941
H	3.844876	-2.088784	6.342480
H	-1.686183	3.244671	6.296144
N	2.697932	-0.513032	-2.594611
N	-3.256655	1.564734	-1.135959
N	4.906251	0.072743	-0.698707
N	-3.061124	-2.220461	-0.171817
N	-0.006789	-0.000158	0.001615
N	3.050652	2.217693	0.181048
N	-4.919142	-0.073837	0.702101
N	3.243124	-1.571475	1.131228
N	-2.708213	0.522225	2.594413
Si	1.845191	-1.377551	-3.844305
Si	-2.786704	2.324970	-2.631495
Si	-2.348195	-3.809666	-0.215820
Si	2.345435	3.810574	0.218950
Si	2.781326	-2.330537	2.630163
Si	-1.847243	1.382528	3.841025
Th	2.108851	0.030876	-0.300317
Th	-2.123924	-0.030613	0.302700

Energy: – 971.45820054 eV

[ThNTh]⁻ start (9⁻) (from Gaussian)

Si	-3.595426000	-0.636881000	-1.088003000
N	-2.283138000	-1.079422000	-2.179320000
C	-1.167472000	-1.655597000	-1.416343000
C	0.017527000	-0.707977000	-1.325935000
N	0.503897000	-0.418323000	-2.670798000
C	0.897527000	0.977835000	-2.861734000
C	-0.265086000	1.770616000	-3.451076000
N	-0.611159000	1.324930000	-4.815528000
Si	0.058909000	2.462951000	-5.986754000
C	-4.055160000	-2.071207000	0.172468000
C	-3.899120000	-3.476440000	-0.417530000
C	-0.819885000	4.193752000	-5.980697000
C	-2.229083000	4.110608000	-6.564493000
Th	-1.742290000	-0.823185000	-4.542846000
N	-3.603320000	-1.014575000	-5.704528000
N	0.131391000	-2.343568000	-5.059853000
C	1.331441000	-1.562896000	-4.661846000
C	1.494282000	-1.376835000	-3.156773000
Si	0.748926000	-3.924597000	-5.582400000
C	1.382920000	-4.980290000	-4.072817000
C	1.991267000	-6.332240000	-4.474628000
C	-3.397822000	-2.034192000	1.562189000
C	0.377618000	-5.177943000	-2.933340000
C	-0.862784000	4.933850000	-4.637289000
Th	-5.589423000	-1.177736000	-6.672825000
N	-5.379943000	-1.304027000	-9.034042000
C	-6.490558000	-0.552099000	-9.639966000
C	-7.826446000	-1.287327000	-9.528527000
N	-8.169276000	-1.553312000	-8.131792000
C	-9.068649000	-0.582754000	-7.506377000
C	-8.485748000	-0.120429000	-6.170457000
N	-7.228299000	0.635843000	-6.326213000
Si	-7.580214000	2.366534000	-6.409929000
N	-6.702510000	-3.307102000	-6.203868000
C	-7.279848000	-3.752625000	-7.482642000
C	-8.520656000	-2.946179000	-7.857288000
C	-8.644770000	3.024229000	-4.895352000
C	-10.173036000	2.860399000	-4.951774000
Si	-7.090335000	-4.350952000	-4.831661000
C	-8.299636000	-5.832789000	-5.251404000
C	-9.684025000	-5.562942000	-5.856561000
Si	-4.211053000	-1.893737000	-10.207557000
C	-5.040011000	-2.804116000	-11.716339000
C	-4.032254000	-3.174243000	-12.816152000
C	-8.482891000	-6.735336000	-4.018339000
C	-8.148418000	2.546808000	-3.530504000
C	-5.926870000	-4.019586000	-11.417360000
H	-3.574320000	-1.098291000	2.099952000
H	-3.791216000	-2.846096000	2.191215000

H	-1.452695000	-1.934849000	-0.390439000
H	-2.312067000	-2.180460000	1.511574000
H	0.821063000	-1.115642000	-0.684972000
H	-0.819145000	-2.596066000	-1.870281000
H	-0.322420000	0.224399000	-0.864953000
H	-0.386668000	-5.917028000	-3.192423000
H	0.872286000	-5.535361000	-2.018763000
H	1.329444000	-2.338680000	-2.661522000
H	-0.144315000	-4.248668000	-2.688170000
H	-4.319443000	-4.236530000	0.256739000
H	2.520388000	-1.049076000	-2.904047000
H	-2.846606000	-3.732635000	-0.577262000
H	1.250067000	1.419171000	-1.912472000
H	-9.117576000	-6.252501000	-3.266605000
H	-4.397932000	-3.577009000	-1.382225000
H	-7.539727000	-6.997541000	-3.530215000
H	-8.707780000	3.026773000	-2.714120000
H	-1.125019000	1.662050000	-2.774186000
H	-8.981923000	-7.675067000	-4.295074000
H	1.224223000	-7.014191000	-4.859924000
H	2.455904000	-6.832529000	-3.612524000
H	2.256013000	-2.014732000	-5.038706000
H	1.736656000	1.017030000	-3.561770000
H	-10.646702000	3.428949000	-4.138084000
H	-8.277843000	1.465332000	-3.419497000
H	-7.088796000	2.758580000	-3.372323000
H	-0.008273000	2.839076000	-3.398726000
H	1.302361000	-0.560168000	-5.114447000
H	-10.301950000	-4.938991000	-5.203212000
H	2.759522000	-6.238795000	-5.248989000
H	-10.227033000	-6.508837000	-5.999366000
H	-1.516314000	4.419719000	-3.923527000
H	-10.479687000	1.816567000	-4.816833000
H	-2.867434000	3.452130000	-5.963828000
H	-10.616056000	3.209603000	-5.889212000
H	-9.638840000	-5.073985000	-6.831945000
H	-1.266848000	5.948774000	-4.762500000
H	0.123811000	5.031787000	-4.169579000
H	-2.707089000	5.099833000	-6.592144000
H	-2.229362000	3.720652000	-7.587403000
H	-8.327012000	-1.024634000	-5.558089000
H	-7.538338000	-4.821671000	-7.486098000
H	-9.208654000	-2.965579000	-7.006428000
H	-9.254231000	0.447257000	-5.624240000
H	-6.557836000	-3.635983000	-8.304390000
H	-9.049730000	-3.397377000	-8.717785000
H	-10.077617000	-1.010403000	-7.372590000
H	-9.174848000	0.285738000	-8.161238000
H	-7.742268000	-2.239418000	-10.060666000
H	-6.604912000	0.431423000	-9.160354000

H	-8.626630000	-0.715028000	-10.029049000
H	-6.323093000	-0.327621000	-10.706101000
H	-5.323937000	-4.911205000	-11.218499000
H	-6.568775000	-3.873644000	-10.545798000
H	-3.395283000	-4.009006000	-12.500912000
H	-3.370663000	-2.345913000	-13.088494000
H	-6.572989000	-4.259273000	-12.273764000
H	-4.545439000	-3.497650000	-13.733595000
C	-4.924393514	-0.218351340	-2.320187350
H	-4.516993263	-0.137244058	-3.334364404
H	-5.706842660	-0.985502001	-2.339180456
H	-5.404642222	0.737050603	-2.080027034
C	-3.219660440	0.754003992	0.088363942
H	-4.109314711	1.046472517	0.657649366
H	-2.445436011	0.468458722	0.809576792
H	-2.862395385	1.642228063	-0.445048049
C	-5.542856581	-1.753807011	0.412501605
H	-6.092718658	-1.680283205	-0.532672665
H	-6.024757143	-2.532175868	1.015014107
H	-5.667497679	-0.802242262	0.941744266
C	1.841627297	2.670006259	-5.498242390
H	1.955272744	2.705573539	-4.408780075
H	2.455406852	1.840400558	-5.867209301
H	2.261525179	3.597302432	-5.904341205
C	-0.174770763	1.799176569	-7.708496511
H	0.786945132	1.648458593	-8.211979464
H	-0.696043513	0.835198344	-7.697172059
H	-0.765605728	2.486681329	-8.324418520
C	0.094217428	5.045421571	-6.881072307
H	1.050831837	5.258671520	-6.390642103
H	0.314042387	4.533335852	-7.824763330
H	-0.371679834	6.006270886	-7.127683351
C	2.198866376	-3.580812062	-6.695541870
H	3.132779345	-3.515814487	-6.125725816
H	2.323382933	-4.371398666	-7.444258099
H	2.073590265	-2.633968717	-7.233038147
C	-0.606888990	-4.879203952	-6.425069141
H	-0.355069232	-5.091637250	-7.470328610
H	-0.787849610	-5.838817171	-5.927572139
H	-1.550324849	-4.321523135	-6.419632494
C	2.503901337	-4.085567786	-3.512038076
H	2.342324916	-3.860991287	-2.451582085
H	3.483112879	-4.569569878	-3.601448782
H	2.557617592	-3.130663344	-4.047183609
C	-5.490716184	-5.156561476	-4.329851262
H	-5.456752485	-5.337414553	-3.249459163
H	-5.355950938	-6.122102074	-4.830528030
H	-4.629961005	-4.528641568	-4.586611426
C	-7.539659637	-6.484686772	-6.421474606
H	-6.488817530	-6.661665631	-6.165510150

H	-7.979998485	-7.450401493	-6.694612517
H	-7.559424155	-5.848720043	-7.313811064
C	-7.912373480	-3.284587636	-3.548415201
H	-8.950945463	-3.063121636	-3.819393983
H	-7.924476034	-3.776422118	-2.569099157
H	-7.390746689	-2.327926869	-3.430880786
C	-8.390228126	4.533437127	-5.065937061
H	-7.748794225	4.922706594	-4.267100228
H	-7.896693937	4.749485193	-6.020324184
H	-9.327338658	5.101244911	-5.042983988
C	-8.592568795	2.660099305	-7.942429304
H	-7.958873009	2.714281594	-8.834955148
H	-9.318516972	1.854609346	-8.101466132
H	-9.151848270	3.600340912	-7.877154033
C	-5.949194354	3.260551215	-6.399530185
H	-5.601523139	3.444531302	-5.376600809
H	-5.173271250	2.681107395	-6.912653698
H	-6.023096478	4.231388000	-6.902643575
C	-3.306596448	-0.463348829	-10.979274582
H	-2.444847024	-0.162185627	-10.372811796
H	-2.934311999	-0.718678261	-11.977933039
H	-3.958540173	0.411388180	-11.083740911
C	-5.956178243	-1.714787911	-12.304218151
H	-5.647636820	-1.438834739	-13.318989303
H	-6.996930179	-2.054213632	-12.356497502
H	-5.936114641	-0.804567198	-11.694132602
C	-3.110854354	-3.053505828	-9.256715687
H	-3.546447948	-3.309270895	-8.284115612
H	-2.950990752	-3.989891834	-9.803279883
H	-2.127057334	-2.607758746	-9.070808091

Energy: –3195.995947 a.u.

Intramolecular TS^{-} (from Gaussian)

Si	-2.925618806	-0.149364475	-0.590383923
C	-4.025279000	-1.605419000	0.517709000
C	-3.266640000	-1.758356000	1.847853000
N	-1.881952806	-0.857106475	-1.821203923
Th	-1.468587806	-1.053670475	-4.234004923
N	0.425425194	-2.482286475	-4.952078923
Si	0.574237000	-4.177773000	-5.741262000
C	-0.904986806	-1.760629475	-1.208592923
C	0.478836194	-1.125402475	-1.118660923
N	0.918032194	-0.758241475	-2.463914923
C	1.877699194	-1.686467475	-3.044477923
C	1.666962194	-1.774695475	-4.546904923
C	1.288297194	0.651952525	-2.626768923
C	0.085631194	1.456191525	-3.116609923
N	-0.344823806	1.029184525	-4.464688923
Si	0.085335194	2.219236525	-5.690892923
C	1.511303000	2.740679000	-5.616744000

C	2.594365000	1.790454000	-6.143692000
C	-4.259543000	-3.005461000	-0.051203000
C	1.760845000	4.151552000	-6.170546000
N	-3.291999806	-1.119266475	-5.524999923
Th	-5.103989806	-1.184450475	-6.807905923
N	-6.156861806	-3.193834475	-6.106013923
Si	-6.550674806	-3.792962475	-4.482900923
C	-7.515890806	-5.486830475	-4.521978923
C	-7.688318806	-6.032959475	-3.093860923
C	0.971777000	-5.419191000	-4.291425000
C	-0.136888000	-5.535138000	-3.239648000
C	1.404378000	-6.818511000	-4.757631000
N	-4.766443806	-1.319583475	-9.226103923
Si	-3.834872000	-1.573095000	-10.544669000
N	-6.903261806	0.448086525	-6.566945923
Si	-7.741124000	2.029607000	-6.615408000
C	-5.996453806	-0.872250475	-9.902768923
C	-7.121038806	-1.884712475	-9.712777923
N	-7.513362806	-1.940097475	-8.308553923
C	-7.868062806	-3.288813475	-7.861610923
C	-6.633910806	-3.955780475	-7.257665923
C	-8.493584806	-0.915466475	-7.943417923
C	-8.158844806	-0.339155475	-6.573586923
C	-8.777985000	2.395391000	-5.000830000
C	-8.074324000	1.832703000	-3.765174000
C	-10.264421000	2.007492000	-4.957413000
C	-4.800500194	-3.232139525	-3.746174077
C	-8.872325806	-5.577678475	-5.234401923
C	-4.379788000	-2.188936000	-12.321148000
C	-5.399259000	-3.332711000	-12.415588000
C	-3.157215000	-2.544518000	-13.184733000
H	-3.191718000	-0.828106000	2.416989000
H	-3.772370000	-2.492165000	2.490216000
H	-1.200204806	-2.107809475	-0.207511923
H	-2.245502000	-2.131925000	1.698879000
H	1.210721194	-1.796446475	-0.636887923
H	-0.800189806	-2.696766475	-1.796481923
H	0.409295194	-0.226087475	-0.498760923
H	-1.020199000	-6.051519000	-3.630179000
H	0.204776000	-6.093587000	-2.356488000
H	1.712658194	-2.678378475	-2.612424923
H	-0.461836000	-4.549263000	-2.897275000
H	-4.816578000	-3.628952000	0.661965000
H	2.918811194	-1.398133475	-2.800711923
H	-3.312522000	-3.521758000	-0.250005000
H	1.685282194	1.065133525	-1.683318923
H	-8.403728806	-5.429479475	-2.522887923
H	-4.816058000	-2.997468000	-0.988205000
H	-6.752297806	-6.056273475	-2.527734923
H	-8.572867000	2.147649000	-2.837184000

H	-0.721869806	1.303692525	-2.391724923
H	-8.081565806	-7.059351475	-3.111827923
H	0.571402000	-7.356351000	-5.226382000
H	1.741656000	-7.433417000	-3.909983000
H	2.565371194	-2.232623475	-4.975912923
H	2.089170194	0.721931525	-3.368707923
H	-10.754693000	2.462020000	-4.084261000
H	-5.296943194	-2.422668525	-3.197688077
H	-8.065326000	0.737285000	-3.784321000
H	-7.031105000	2.156085000	-3.701517000
H	0.331312194	2.527286525	-3.063646923
H	1.662039194	-0.748135475	-4.939123923
H	-9.628998806	-4.947128475	-4.758695923
H	2.229331000	-6.787574000	-5.479046000
H	-9.249231806	-6.610783475	-5.198741923
H	-3.942946194	-3.507378525	-3.115326077
H	-10.395564000	0.924835000	-4.852658000
H	-10.824248000	2.322885000	-5.843654000
H	-3.709118806	-2.389563475	-4.462585923
H	2.466304000	0.766235000	-5.781138000
H	-8.827984806	-5.298945475	-6.289943923
H	-8.102349806	-1.174195475	-5.864661923
H	2.588268000	1.746939000	-7.238554000
H	-6.860004806	-5.003567475	-7.014970923
H	3.598119000	2.124743000	-5.843222000
H	-8.626667806	-3.210714475	-7.075775923
H	-9.027588806	0.248538525	-6.244048923
H	-5.861813806	-4.022178475	-8.043463923
H	1.089990000	4.900166000	-5.738472000
H	2.790147000	4.475565000	-5.960189000
H	-8.308333806	-3.880253475	-8.684253923
H	1.635270000	4.190574000	-7.258109000
H	-9.518107806	-1.330096475	-7.958435923
H	-8.459244806	-0.114030475	-8.684899923
H	-6.726759806	-2.866897475	-9.988358923
H	-6.342407806	0.103308525	-9.522779923
H	-7.986517806	-1.672356475	-10.368253923
H	-5.862846806	-0.715722475	-10.985237923
H	-5.120481000	-4.196476000	-11.804864000
H	-6.398762000	-3.021022000	-12.104179000
H	-2.735675000	-3.511396000	-12.888381000
H	-2.350041000	-1.806696000	-13.123094000
H	-5.485804000	-3.685269000	-13.454107000
H	-3.438661000	-2.632810000	-14.244022000
C	-6.555769289	-6.441994413	-5.255074091
H	-5.691964304	-6.696467895	-4.630420068
H	-7.054950719	-7.379367050	-5.525736211
H	-6.174078675	-5.992607603	-6.178909812
C	-7.861647069	-3.261225493	-3.275338292
H	-8.836280144	-3.155688887	-3.765298340

H	-7.976830057	-3.989939408	-2.464896159
H	-7.615547171	-2.295831597	-2.818656635
C	-5.406168336	-1.025913771	0.876760989
H	-6.024060575	-0.884014017	-0.017208862
H	-5.953897503	-1.690607500	1.554464869
H	-5.313927986	-0.052662680	1.372122174
C	-8.726969842	3.931606274	-4.905750637
H	-9.608955975	4.329021563	-4.390706947
H	-7.842453879	4.268023570	-4.352973595
H	-8.689845463	4.391891848	-5.899665946
C	1.692584302	2.832231098	-4.090193869
H	0.726047152	2.847061357	-3.573769183
H	2.260165979	1.978017142	-3.703829922
H	2.232451842	3.742780309	-3.806377301
C	-5.035280742	-0.953566679	-12.965967282
H	-4.494425289	-0.638795793	-13.865691196
H	-6.071379924	-1.158596866	-13.258488479
H	-5.049558612	-0.103039770	-12.274947174
C	-8.932472795	2.204809647	-8.033008819
H	-8.407193250	2.233527927	-8.994447904
H	-9.637951625	1.366814745	-8.067239027
H	-9.519002475	3.126844298	-7.949679903
C	-6.334779170	3.233078967	-6.798299889
H	-5.895735238	3.484464768	-5.826109548
H	-5.535586919	2.820673814	-7.424655442
H	-6.667919681	4.168630346	-7.261840273
C	-1.033627276	3.665452575	-5.350331439
H	-1.577492720	3.970103726	-6.251722869
H	-1.777038796	3.422646525	-4.582547565
H	-0.466492158	4.534364763	-4.997545329
C	-0.080904869	1.417791354	-7.361117423
H	0.899485778	1.237822080	-7.816698034
H	-0.594547717	0.452200911	-7.291019889
H	-0.654641599	2.047557372	-8.050558935
C	2.181185489	-4.775399037	-3.588244357
H	1.984726523	-4.616681377	-2.521791811
H	3.072793293	-5.407542104	-3.669103553
H	2.426123642	-3.801545704	-4.027247385
C	1.893838583	-3.893485493	-7.020883926
H	2.888458774	-3.841300528	-6.563592050
H	1.912784694	-4.701655621	-7.760890435
H	1.729542212	-2.954537405	-7.561709093
C	-0.905704171	-4.922766687	-6.586459337
H	-0.713906575	-5.090749182	-7.652341139
H	-1.174214520	-5.888449449	-6.143213876
H	-1.781453258	-4.268564032	-6.507775840
C	-1.819266370	0.894887182	0.479720167
H	-2.287949121	1.111536449	1.446419721
H	-0.866838278	0.391104508	0.680197817
H	-1.590985101	1.854081573	0.001240853

C	-4.257173775	0.885576562	-1.374895499
H	-4.028976523	1.100198399	-2.425120206
H	-5.227434009	0.376829068	-1.345254156
H	-4.372073321	1.845771072	-0.859208317
C	-3.433650341	0.121180311	-11.198926098
H	-2.473226317	0.480972654	-10.812605483
H	-3.370397718	0.121747388	-12.293049641
H	-4.199108971	0.851224102	-10.912189598
C	-2.746737013	-2.865447708	-9.766602983
H	-3.112019757	-3.149097931	-8.773013771
H	-2.709053787	-3.775213386	-10.376532428
H	-1.718834311	-2.503764277	-9.649514115

Energy: −3195.969811 a.u

Final product$^-$ (8$^-$) (from Gaussian)

Si	-4.432295236	-0.543499119	-1.354093273
N	-2.790033376	-1.126978866	-1.621380559
C	-1.767233800	-1.660899230	-0.711637612
C	-0.676890037	-0.645033545	-0.412393080
N	-0.011977114	-0.274166226	-1.657166129
C	0.310557294	1.150204245	-1.745475866
C	-0.799688869	1.890552314	-2.484889748
N	-0.902696633	1.476643928	-3.898467139
Si	-0.140662657	2.698448738	-4.919422814
C	-1.125441134	4.369265085	-5.000489252
C	-2.420258678	4.220387383	-5.797265668
Th	-1.913860451	-0.747005481	-3.864675088
N	-3.558746464	-1.010019697	-5.305071348
N	0.115477127	-2.126047654	-4.128441794
C	1.185094227	-1.287517849	-3.527804324
C	1.103609754	-1.149403289	-2.011009918
Si	0.911232116	-3.644087237	-4.593616346
C	1.377821956	-4.715016216	-3.034551889
C	2.130577873	-6.009796065	-3.375448686
C	0.226318991	-5.018787464	-2.070253904
C	-1.422711968	5.053217907	-3.659531006
Th	-5.656560455	-1.048762531	-5.493681138
N	-5.857150324	-1.119614378	-7.857982458
C	-7.069700352	-0.380124761	-8.243835829
C	-8.352213074	-1.147542971	-7.921646415
N	-8.442813496	-1.451098581	-6.493782750
C	-9.238010716	-0.514729152	-5.698250085
C	-8.441076481	-0.068125995	-4.472019408
N	-7.243872166	0.719505944	-4.823762231
Si	-7.636961681	2.443209217	-4.802047392
N	-6.631904207	-3.212282023	-4.892872620
C	-7.413768268	-3.643191555	-6.063376109
C	-8.715447485	-2.857110366	-6.197640219
C	-8.434926286	3.044065631	-3.110299852
C	-9.946545525	2.847026254	-2.905840959

Si	-6.756655563	-4.293814973	-3.500857290
C	-7.992746144	-5.793228444	-3.742255941
C	-9.465818854	-5.541782663	-4.092108927
Si	-4.898601093	-1.657479450	-9.230237430
C	-5.959485591	-2.553765846	-10.595367164
C	-5.150897193	-2.877372767	-11.861708415
C	-7.942788989	-6.725924301	-2.519061149
C	-7.700798926	2.548804261	-1.864154044
C	-6.758446746	-3.795079576	-10.178143067
H	-2.185904092	-1.996568725	0.249535647
H	0.045325572	-1.025787888	0.333344922
H	-1.290813052	-2.559609372	-1.132608748
H	-1.145573141	0.245860855	0.016601896
H	-0.437638874	-5.793852913	-2.465142790
H	0.598879866	-5.379268864	-1.100642281
H	0.930818577	-2.137951039	-1.574538292
H	-0.388691927	-4.134125064	-1.882614833
H	2.054582022	-0.767900005	-1.593521109
H	0.483525672	1.576082963	-0.741050897
H	-8.446425386	-6.273775864	-1.657103572
H	-6.924611714	-6.977181603	-2.207999038
H	-8.119008068	2.998397663	-0.951628992
H	-1.743387777	1.702456994	-1.952438873
H	-8.464737828	-7.670528634	-2.728826764
H	1.479265196	-6.723220289	-3.893796392
H	2.491216484	-6.512634790	-2.466332114
H	2.184796227	-1.665751809	-3.769770428
H	1.241976594	1.268698662	-2.305958952
H	-10.282477751	3.387153985	-2.008560863
H	-7.788976294	1.462549957	-1.759559300
H	-6.633903826	2.781000436	-1.886226073
H	-0.626915399	2.970216405	-2.363337336
H	1.157411444	-0.271984758	-3.950986270
H	-9.972615527	-4.946097923	-3.326388420
H	2.999767137	-5.838793217	-4.018760990
H	-10.007746187	-6.496413577	-4.162556719
H	-2.141017034	4.472176840	-3.070194557
H	-10.205785760	1.793883596	-2.746109325
H	-3.096568386	3.500672579	-5.321346036
H	-10.551711731	3.206168599	-3.743570509
H	-9.599416546	-5.031120388	-5.048074552
H	-1.870855602	6.044872170	-3.817028123
H	-0.527881962	5.194809029	-3.042323416
H	-2.954436610	5.178040941	-5.870573337
H	-2.238161290	3.870648391	-6.818506975
H	-8.161899678	-0.981444724	-3.919129203
H	-7.649102076	-4.717480124	-6.048869439
H	-9.245100771	-2.910283143	-5.241439194
H	-9.113650923	0.470220359	-3.787173915
H	-6.847377355	-3.492680569	-6.994356365

H	-9.377193078	-3.301574851	-6.964776525
H	-10.200430513	-0.967817005	-5.402854443
H	-9.472157311	0.364991361	-6.302953899
H	-8.343911149	-2.085857393	-8.483991724
H	-7.117401540	0.590050951	-7.727208576
H	-9.237490274	-0.582848480	-8.261862910
H	-7.093718693	-0.129106223	-9.316911572
H	-6.113766039	-4.676921198	-10.108857565
H	-7.242186963	-3.682394101	-9.205384719
H	-4.453583764	-3.704113967	-11.682291165
H	-4.561970126	-2.028667865	-12.223465660
H	-7.538653808	-4.030865610	-10.915726497
H	-5.809232507	-3.192528033	-12.684462547
C	-5.535980967	-0.259653597	-3.230327945
H	-6.327857561	-1.003648861	-3.373541245
H	-6.019216163	0.714186379	-3.091662454
C	-4.404108593	0.927306042	-0.215876383
C	1.528561233	2.997962654	-4.155496758
H	1.472130190	2.998826124	-3.060999957
H	2.246242995	2.223198562	-4.448380752
H	1.941590534	3.964625620	-4.465461557
C	-0.063598930	2.087770651	-6.674624642
H	0.971607033	2.017019388	-7.027401237
H	-0.513917351	1.093292449	-6.771237104
H	-0.598889806	2.759980462	-7.354849840
C	-0.144660484	5.310411975	-5.724306804
H	0.709325579	5.564255296	-5.086043670
H	0.250662711	4.849633543	-6.636726856
H	-0.631597981	6.248912354	-6.012781382
C	2.487216770	-3.167720346	-5.459033559
H	3.316772519	-3.066192483	-4.750064021
H	2.777733717	-3.919688942	-6.201496135
H	2.381417067	-2.210698133	-5.982506174
C	-0.232389156	-4.648812379	-5.662380948
H	0.189641298	-4.804852149	-6.661705890
H	-0.421570701	-5.636230872	-5.226121156
H	-1.201251028	-4.151974932	-5.787152322
C	2.336931989	-3.773934555	-2.282173491
H	2.000740033	-3.600710575	-1.253544853
H	3.348871956	-4.191833595	-2.232722115
H	2.406710888	-2.797744933	-2.775421296
C	-5.079635069	-5.073687957	-3.303372128
H	-4.855608124	-5.276861494	-2.249977189
H	-5.015818610	-6.024978374	-3.843817819
H	-4.288180127	-4.421134254	-3.689223668
C	-7.435103344	-6.402555836	-5.042007701
H	-6.352708024	-6.561217681	-4.976013433
H	-7.898152747	-7.371883508	-5.259025344
H	-7.620986846	-5.748202704	-5.901296926
C	-7.363473124	-3.274050640	-2.068567810

H	-8.437212032	-3.070324180	-2.150352536
H	-7.196564603	-3.786926541	-1.114519908
H	-6.847178568	-2.308599776	-2.019037707
C	-8.241793617	4.561946991	-3.284472039
H	-7.478946710	4.948372028	-2.599016904
H	-7.925211163	4.809503336	-4.304079291
H	-9.171099408	5.107832700	-3.085705314
C	-8.904843619	2.746652012	-6.128696502
H	-8.436526361	2.834258140	-7.115667913
H	-9.632324485	1.928576532	-6.179996021
H	-9.461664196	3.672412262	-5.944231123
C	-6.045660511	3.373388468	-5.051300571
H	-5.529442581	3.543185401	-4.099567150
H	-5.359806929	2.822748080	-5.705141201
H	-6.223601881	4.352878152	-5.509607781
C	-4.168218371	-0.190814089	-10.110597470
H	-3.220134935	0.116647323	-9.654841250
H	-3.969972221	-0.416194092	-11.164641995
H	-4.844488276	0.671022555	-10.078891239
C	-6.983685436	-1.473037371	-10.988610904
H	-6.860838468	-1.168485739	-12.034204219
H	-8.011282202	-1.834671020	-10.868721226
H	-6.875057511	-0.575891765	-10.368579931
C	-3.628980023	-2.812245866	-8.513176161
H	-3.884616787	-3.098582673	-7.486643388
H	-3.548941658	-3.732844955	-9.102411870
H	-2.636277558	-2.348494502	-8.488986130
H	-3.160665423	-1.171639188	-6.208072247
C	-5.828571644	1.423427781	0.094555956
H	-6.417796819	1.543542722	-0.821683395
H	-6.364180701	0.719372806	0.741500369
H	-5.810988877	2.393009988	0.605153509
C	-3.729467084	0.585319041	1.125579440
H	-4.467421614	0.488024276	1.929983129
H	-3.179590695	-0.360821975	1.065908026
H	-3.016982113	1.362925302	1.423576655
C	-3.628796438	2.103282450	-0.838428028
H	-2.783545563	2.402231583	-0.208119509
H	-3.228599820	1.840126228	-1.824175974
H	-4.271585325	2.981590390	-0.966904793
C	-5.419217266	-1.853661645	-0.477133147
H	-6.304228217	-2.141049045	-1.056160394
H	-4.823731516	-2.759002364	-0.313235477
H	-5.764856509	-1.504128058	0.502391461

Energy: −3196.027500 a.u

References

1. D. Patel, A. J. Wooles, E. Hashem, H. Omorodion, R. J. Baker, S. T. Liddle, *New J. Chem.,* 2015, **39**, 7559.
2. T. Cantat, B. L. Scott, J. L. Kiplinger, *Chem. Comm.,* 2010, **46**, 919.
3. B. M. Gardner, W. L. Lewis, A. J. Blake, S. T. Liddle, *Organometallics,* 2015, **34**, 2386.
4. B. M. Gardner, J. McMaster, W. Lewis, A. J. Blake, S. T. Liddle, *J. Am. Chem. Soc.,* 2009, **131**, 10388.
5. D. E. Bergbreiter, J. M. Killough, *J. Am. Chem. Soc.,* 1978, **100**, 2126.
6. P. B. Hitchcock, M. F. Lappert, L. Maron, A. V. Protchenko, *Angew. Chem. Int. Ed.,* 2008, **47**, 1488.
7. C. Fonseca Guerra, J. G. Snijders, G. Te Velde, E. J. Baerends, *Theor. Chem. Acc.,* 1998, **99**, 391.
8. G. Te Velde, F. M. Bickelhaupt, S. J. van Gisbergen, A. C. Fonseca Guerra, E. J. Baerends, J. G. Snijders, T. Ziegler, *J. Comput. Chem.,* 2001, **22**, 931.
9. S. H. Vosko, L. Wilk, M. Nusair, *Can. J. Phys.,* 1980, **58**, 1200.
10. A. D. Becke, *Phys. Rev. A.,* 1988, **38**, 3098.
11. J. P. Perdew, *Phys. Rev. B.,* 1986, **33**, 8822.
12. S. Portmann, H. P. Luthi, *Chimia,* 2000, **54**, 766.
13. R. F. W. Bader, *Atoms in Molecules: A Quantum Theory*, Oxford University Press, New York, 1990.
14. R. F. W. Bader, *J. Phys. Chem. A,* 1998, **102**, 7314.
15. http://www.quimica.urv.es/XAIM.
16. Gaussian 09, Revision E.01, M. J. Frisch, G. W. Trucks, H. B. Schlegel, G. E. Scuseria, M. A. Robb, J. R. Cheeseman, G. Scalmani, V. Barone, B. Mennucci, G. A. Petersson,

H. Nakatsuji, M. Caricato, X. Li, H. P. Hratchian, A. F. Izmaylov, J. Bloino, G. Zheng, J. L. Sonnenberg, M. Hada, M. Ehara, K. Toyota, R. Fukuda, J. Hasegawa, M. Ishida, T. Nakajima, Y. Honda, O. Kitao, H. Nakai, T. Vreven, J. A. Montgomery, Jr.; J. E. Peralta, F. Ogliaro, M. Bearpark, J. J. Heyd, E. Brothers, K. N. Kudin, V. N. Staroverov, R. Kobayashi, J. Normand, K. Raghavachari, A. Rendell, J. C. Burant, S. S. Iyengar, J. Tomasi, M. Cossi, N. Rega, J. M. Millam, M. Klene, J. E. Knox, J. B. Cross, V. Bakken, C. Adamo, J. Jaramillo, R. Gomperts, R. E. Stratmann, O. Yazyev, A. J. Austin, R. Cammi, C. Pomelli, J. W. Ochterski, R. L. Martin, K. Morokuma, V. G. Zakrzewski, G. A. Voth, P. Salvador, J. J. Dannenberg, S. Dapprich, A. D. Daniels, Ö. Farkas, J. B. Foresman, J. V. Ortiz, J. Cioslowski and D. J. Fox, Gaussian, Inc., Wallingford CT, 2009.

17. A. D. Becke, *J. Chem. Phys*., 1993, 98, 5648.
18. K. Burke, J. P. Perdew, W. Yang, Electronic Density Functional Theory: Recent Progress and New Directions; Plenum: New York, 1998.
19. W. Kuchle, M. Dolg, H. Stoll, H. Preuss, *J. Chem. Phys*., 1994, **100**, 7535.
20. X. Y. Cao, M. Dolg, H. Stoll, *J. Chem. Phys*., 2003, **118**, 487.
21. X. Y. Cao, M. Dolg, *J. Molec. Struc. (Theochem),* 2004, **673**, 203.
22. P. C. Hariharan, J. A. Pople, *Theor. Chim. Acta,* 1973, **28**, 213.
23. W. J. Hehre, R. Ditchfield, J. A. Pople, *J. Chem. Phys.,* 1972, **56**, 2257.
24. B. M. Gardner, P. A. Cleaves, C. E. Kefalidis, J. Fang, L. Maron, W. Lewis, A. J. Blake, S. T. Liddle, *Chem. Sci.,* 2014, **5**, 2489.
25. L. Castro, E. Kirillov, O. Miserque, A. Welle, L. Haspeslagh, J. -F. Carpentier, L. Maron, *ACS Catalysis*, 2015, **5**, 416.
